# A Model for the Epigenetic Switch Linking Inflammation to Cell Transformation: Deterministic and Stochastic Approaches

**DOI:** 10.1371/journal.pcbi.1003455

**Published:** 2014-01-30

**Authors:** Claude Gérard, Didier Gonze, Frédéric Lemaigre, Béla Novák

**Affiliations:** 1 Oxford Centre for Integrative Systems Biology, Department of Biochemistry, University of Oxford, Oxford, United Kingdom; 2 de Duve Institute, Université Catholique de Louvain (UCL), Brussels, Belgium; 3 Faculté des Sciences, Université Libre de Bruxelles (ULB), Campus Plaine, Brussels, Belgium; École Polytechnique Fédérale de Lausanne, Switzerland

## Abstract

Recently, a molecular pathway linking inflammation to cell transformation has been discovered. This molecular pathway rests on a positive inflammatory feedback loop between NF-κB, Lin28, Let-7 microRNA and IL6, which leads to an epigenetic switch allowing cell transformation. A transient activation of an inflammatory signal, mediated by the oncoprotein Src, activates NF-κB, which elicits the expression of Lin28. Lin28 decreases the expression of Let-7 microRNA, which results in higher level of IL6 than achieved directly by NF-κB. In turn, IL6 can promote NF-κB activation. Finally, IL6 also elicits the synthesis of STAT3, which is a crucial activator for cell transformation. Here, we propose a computational model to account for the dynamical behavior of this positive inflammatory feedback loop. By means of a deterministic model, we show that an irreversible bistable switch between a transformed and a non-transformed state of the cell is at the core of the dynamical behavior of the positive feedback loop linking inflammation to cell transformation. The model indicates that inhibitors (tumor suppressors) or activators (oncogenes) of this positive feedback loop regulate the occurrence of the epigenetic switch by modulating the threshold of inflammatory signal (Src) needed to promote cell transformation. Both stochastic simulations and deterministic simulations of a heterogeneous cell population suggest that random fluctuations (due to molecular noise or cell-to-cell variability) are able to trigger cell transformation. Moreover, the model predicts that oncogenes/tumor suppressors respectively decrease/increase the robustness of the non-transformed state of the cell towards random fluctuations. Finally, the model accounts for the potential effect of competing endogenous RNAs, ceRNAs, on the dynamics of the epigenetic switch. Depending on their microRNA targets, the model predicts that ceRNAs could act as oncogenes or tumor suppressors by regulating the occurrence of cell transformation.

## Author Summary

An increasing amount of evidence demonstrates a close relation between inflammation and cancer development, which reveals the importance of the tumor microenvironment for the development of cancers. Recently, a molecular pathway linking inflammation to cell transformation, which is a prerequisite to cancer development, has been discovered. This molecular pathway is based on a positive inflammatory feedback loop between NF-κB, Lin28, Let-7 microRNA and IL6, allowing the occurrence of an epigenetic switch leading to cell transformation. Here, we propose a computational model to account for the dynamics of this epigenetic switch. We show that an irreversible bistable switch is at the core of the dynamics of the system. The model further indicates that oncogenes (activators of the switch) and tumor suppressors (inhibitors of the switch) regulate the occurrence of cell transformation by modulating the threshold of inflammatory signal needed to induce the switch. Stochastic simulations of the model suggest that molecular fluctuations are able to trigger cell transformation, highlighting possible links between stochasticity and cancer development. Finally, the model predicts a crucial role of competing endogenous RNAs (ceRNAs) for the dynamics of the epigenetic switch and the occurrence of cell transformation.

## Introduction

The characteristics of cancer rest on many biological capabilities acquired during the multistep of the development of tumors [1]. These biological properties include sustaining proliferative signaling, evading growth suppressors, resisting cell death, allowing replicative immortality, promoting angiogenesis, and eliciting formation of metastasis [1]. The progression from normal cells to cancer could be strongly influenced by the tumor microenvironment. In that context, many studies have shown close relations between inflammation and different types of cancer [2]–[4]. Inflammatory molecules, such as the interleukin-6 (IL6) or the transcription factor NF-κB, could provide growth signals, which elicit the proliferation of malignant cells [5], [6]. However, until recently, the molecular regulatory network linking inflammation to cell transformation was poorly understood.

To study the molecular link between inflammation and cancer, Iliopoulos and coworkers used an experimental model of oncogenesis, which involves a derivative of MCF10A, a spontaneous immortalized cell line derived from normal mammary epithelial cells containing ER-Src, a fusion of the oncoprotein Src with the ligand binding domain of estrogen receptor [7]. They demonstrated that transient treatment with tamoxifen results in stable cell transformation, defined by their invasive capabilities, their increased motility, as well as their ability to form mammospheres (multicellular structure enriched in cancer stem cells). This stable cell transformation can be defined as an epigenetic switch, which corresponds to a stable cell change to another phenotype without any change in DNA sequence. The triggering event of the epigenetic switch is mediated by a transient inflammatory signal driven by the Src oncoprotein [7]. After the triggering event, the transformed state of the cell mediated by the epigenetic switch is stable for many generations. A positive inflammatory feedback loop driven by the transcription factor NF-κB, the microRNA binding protein Lin28, the Let-7 microRNA, and IL6 is responsible for the maintenance of this transformed state [7].

Besides the critical role of microRNAs in the regulation of protein expression [8]–[10] or in conferring robustness of biological processes [11]–[14]; microRNAs, such as miR-122, are at the core of molecular regulatory feedback loop driving hepatocyte differentiation [15], or microRNAs, such as Let-7 microRNA family, are critical regulators in cancer development and progression [16]–[19]. The positive inflammatory feedback loop discovered by Iliopoulos and coworkers brings to light the importance of microRNAs in the molecular regulatory circuit linking inflammation to cell transformation [7], [20].

Other studies showed that epigenetic regulations could play critical role in cancer [21]–[24], and open novel perspectives in understanding developmental processes and diseases [25].

Here, we propose a computational model of a large network involving kinases, transcription factors, messenger RNAs and miRNAs to account for the qualitative dynamics of the epigenetic switch linking inflammation to cell transformation [7], [20]. By means of a deterministic model for the epigenetic switch, we will study the dynamical nature of the positive inflammatory feedback loop leading to cell transformation. We will see how tumor suppressors (inhibitors of this positive feedback loop) or oncogenes (activators of the inflammatory feedback loop) regulate the occurrence of cell transformation. By resorting to stochastic simulations as well as deterministic simulations in a heterogeneous cell population, we will assess the role of random fluctuations (resulting from molecular noise or from cell-to-cell variability) on the dynamics of the epigenetic switch. Furthermore, based on recent experiments describing the potential crucial role of competing endogenous RNA (ceRNA) as microRNA sponge on the control of molecular regulatory network [26]–[29], we will study the effect of ceRNAs on the dynamical behavior of the model for the epigenetic switch linking inflammation to cell transformation.

## Results

### Model for the epigenetic switch linking inflammation to cell transformation

**Figure 1:**
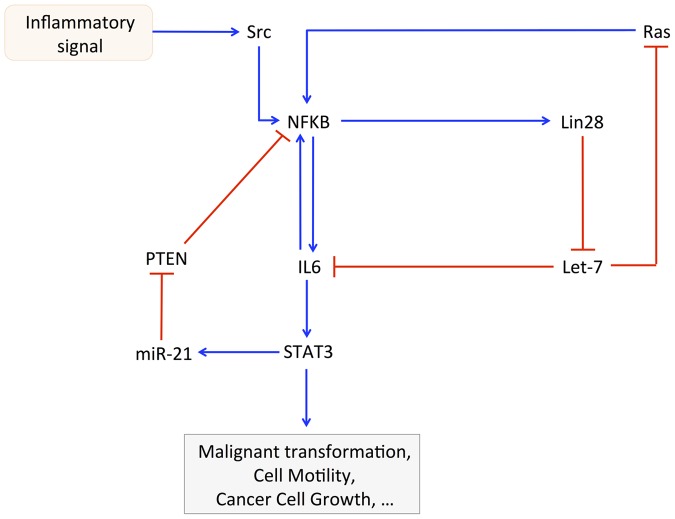
Wiring diagram of the model for the epigenetic switch linking inflammation to cell transformation [7], [20]. The Src oncoprotein elicits an inflammatory response regulated by NF-κB, which activates the transcription of Lin28. The latter protein represses the synthesis of Let-7 microRNA, which prevents Let-7 from inhibiting the expression of IL6. Since IL6 activates NF-κB, this creates a positive feedback loop with a higher activation of IL6 than achieved by NF-κB alone (see mutual activation between NF-κB and IL6). Furthermore, two other positive feedback loops are considered in the model: (1) the oncogene Ras activates NF-κB, which directly promotes the synthesis of Lin28, resulting in an inhibition of the synthesis of Let-7. The down-regulation of Let-7 prevents it from inhibiting the translation of Ras. (2) NF-κB activates IL6, which promotes the transcription of STAT3. The latter transcription factor elicits the synthesis of miR-21, which inhibits the translation of the tumor suppressor PTEN. The down-regulation of PTEN prevents it from inhibiting NF-κB. For the sake of simplicity, we consider in the model that PTEN directly down-regulates the activity of NF-κB. Actually, PTEN down-regulates Akt, which activates NF-κB [20], [36], [37]. For clarity reasons, the inhibitory complexes between Let-7 microRNA and IL6 mRNA, between Let-7 and Ras mRNA, as well as between miR-21 microRNA and PTEN mRNA are not represented in this scheme. All variables included in the model together with their definition are listed in Table 1.

We propose a computational model for the epigenetic switch linking inflammation to cell transformation (see Fig. 1 as well as [7], [20]). The model is based on a positive inflammatory feedback loop between NF-κB, Lin28, Let-7 microRNA and IL6. An inflammatory signal mediated by the oncoprotein Src activates NF-κB, which promotes the synthesis of Lin28. The microRNA-binding protein Lin28 rapidly reduces the synthesis of mature Let-7 microRNA, which ensures a higher production of IL6 than achieved directly by NF-κB. In turn, IL6 triggers the activation of NF-κB and promotes the synthesis of the transcription factor STAT3, whose activity is crucial to induce cell transformation [30]–[35]. As shown by Iliopoulos and coworkers [20], STAT3 is not only a downstream output of the inflammatory regulatory signal, but is part of the positive feedback loop linking inflammation to cancer. Indeed, STAT3 triggers the synthesis of miR-21 and miR-181b-1 microRNAs [20]. For simplicity, we only consider miR-21 in the model (see Fig. 1). The latter microRNA down-regulates the translation of the tumor suppressor PTEN, which is responsible for the inhibition of the activity of NF-κB (see Fig. 1 as well as [20], [36], [37]). Furthermore, we also consider the positive feedback loop between NF-κB, Lin28, Let-7 and the oncoprotein Ras. Indeed, NF-κB activates the synthesis of Lin28, which inhibits the synthesis of Let-7. The down-regulation of Let-7 prevents it to repress the synthesis of Ras, which promotes the activation of NF-κB (see Fig. 1).

In healthy cells, negative feedback loops between NF-κB and IκB protein family control NF-κB oscillations and ensure the occurrence of a transient inflammatory response within the cell [38]–[42]. However in the immortalized cell line [7], it seems that NF-κB is rapidly activated through I-κBα phosphorylation, but its activity remains elevated during all the cell transformation process. For that reason and in order to confine our model to the dynamics of the epigenetic switch, we do not consider negative feedback regulation between NF-κB and IκB.

The model proposed for the molecular mechanisms of the epigenetic switch, linking inflammation to a stable cell transformation, counts 14 kinetic equations describing the time evolution of the concentration of the different variables considered (see Section “Methods” here below, as well as Table 1 for a definition of the different variables of the model).

**Table 1:** Variables of the model.

Symbol	Definition
NFKB	Transcription factor NF-κB
Lin28	MicroRNA binding protein Lin28
Let7	MicroRNA family Let-7, which represses the synthesis of IL6 and Ras
mIL6	Messenger RNA of IL6
mIL6Let7	Complex form between mIL6 and Let7
IL6	Interleukin 6 protein, which promotes the epigenetic switch leading to cell transformation
mRas	Messenger RNA of Ras oncogene
mRasLet7	Complex form between mRas and Let7
Ras	Protein form of Ras oncogene
STAT3	Transcription factor STAT3, whose activity is crucial to promote cell transformation
miR21	MicroRNA miR21, which represses the translation of the tumor suppressor PTEN
mPTEN	Messenger RNA of the tumor suppressor PTEN
miRmpten	Complex form between miR21 and mPTEN
PTEN	Protein form of the tumor suppressor PTEN (phosphatase and tensin homologue)
**Addition of a competing endogenous RNA (ceRNA), which binds to Let-7 microRNA**	
ceRNA	Competing endogenous RNA, which can bind to Let7
ceRNAlet7	Complex between ceRNA and Let7
**Addition of PTEN1 mRNA, which binds to the microRNA miR-21**	
mPTEN1	Messenger RNA of PTEN1
miRmpten1	Complex between mPTEN1 and miR21

**Figure 2:**
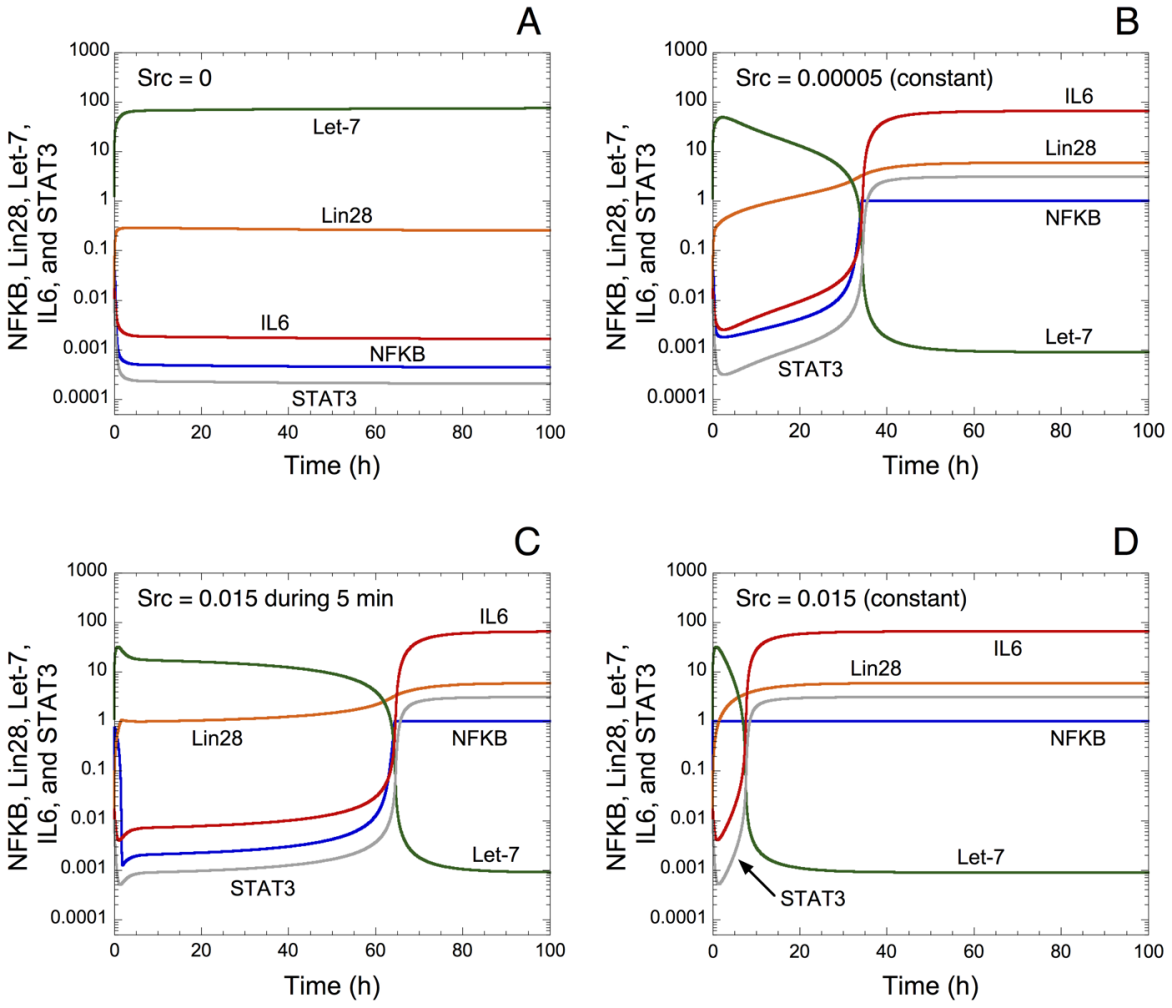
Activation of the epigenetic switch by the Src oncoprotein. Time evolution of NF-κB, Lin28, Let-7, IL6 and STAT3 in the absence of Src in A; in the presence of a constant, low level of Src (Src = 0.00005) in B; in the presence of a transient, higher level of Src (Src = 0.015 during 5 min from t = 0) in C; and in the presence of a constant, higher level of Src (Src = 0.015) in D. (A) The absence of Src prevents the occurrence of an inflammatory response. Let-7 is present at a high level, while low levels of NF-κB, Lin28, IL6 and STAT3 are observed. (B) The presence of Src generates an inflammatory response: a switch leading to cell transformation characterized by a low level of Let-7 together with high levels of NF-κB, Lin28, IL6 and STAT3 occurs at t = 36 h. (C) 5 min of Src activation is sufficient to turn on the switch after t = 60 h. These time scales correspond to those observed in the experiments [7]. (D) The model predicts that, with a constant high level of Src, the switch occurs earlier. Parameter values used in the simulations are given in Table 2. Values for the initial conditions used can be found in the Section: “Methods”.

As observed in the experiments [7], a transient inflammatory signal mediated by Src is sufficient to trigger the switch from an immortalized cell line to a stable transformed state (see Fig. 2). Time evolution of Let-7, Lin28, IL6, NF-κB and STAT3 is shown in the absence of Src (Fig. 2A), in the presence of a constant, low level of Src (Fig. 2B), in the presence of higher level of Src for only 5 min (Fig. 2C), or in the presence of a constant higher level of Src (Fig. 2D). In the absence of Src, a high level of Let-7 together with low levels of Lin28, IL6, NF-κB and STAT3 are observed (Fig. 2A). We define those expression levels as a normal, non-transformed state of the cell. However, with a constant level of Src (Fig. 2B, D) or with a transient level of Src for only 5 min (Fig. 2C), the model exhibits a switch-like behavior in the expression levels of Let-7, Lin28, IL6, NF-κB and STAT3. Indeed, the level of Let-7 is reduced while levels of Lin28, IL6, NF-κB and STAT3 are highly increased at about t = 35 h (Fig. 2B), t = 65 h (Fig. 2C), and t = 10 h (Fig. 2D). In the model, we define this switch as the passage to cell transformation. As in the experiments [7], the model shows that cell transformation can occur with an inflammatory signal of only 5 min. In the latter case, the occurrence of cell transformation is slow, and happens at t = 65 h instead of t = 36 h with a constant level of Src (compare Fig. 2B and 2C as well as [7]). The model is characterized by a biphasic regulation of the different variables. For instance, we can observe a first slow increase of IL6 followed by a boost of IL6 expression. This dynamical behavior may correspond to the experimental observation showing also a biphasic regulation of IL6 expression [7].

Furthermore, the model also accounts for the experimental observations showing that the positive feedback loop involving NF-κB, Lin28, Let-7, and IL6 is required for maintenance of the transformed state of the cell [7]. Indeed, from a stable transformed state, a transient inhibition of either NF-κB, Lin28, or IL6 (as in the experiments) abolishes the transformed state and brings back the normal, non-transformed state of the cell (see Fig. S1).

### Effect of the tumor suppressors, Let-7 & PTEN, on the dynamics of cell transformation

**Figure 3:**
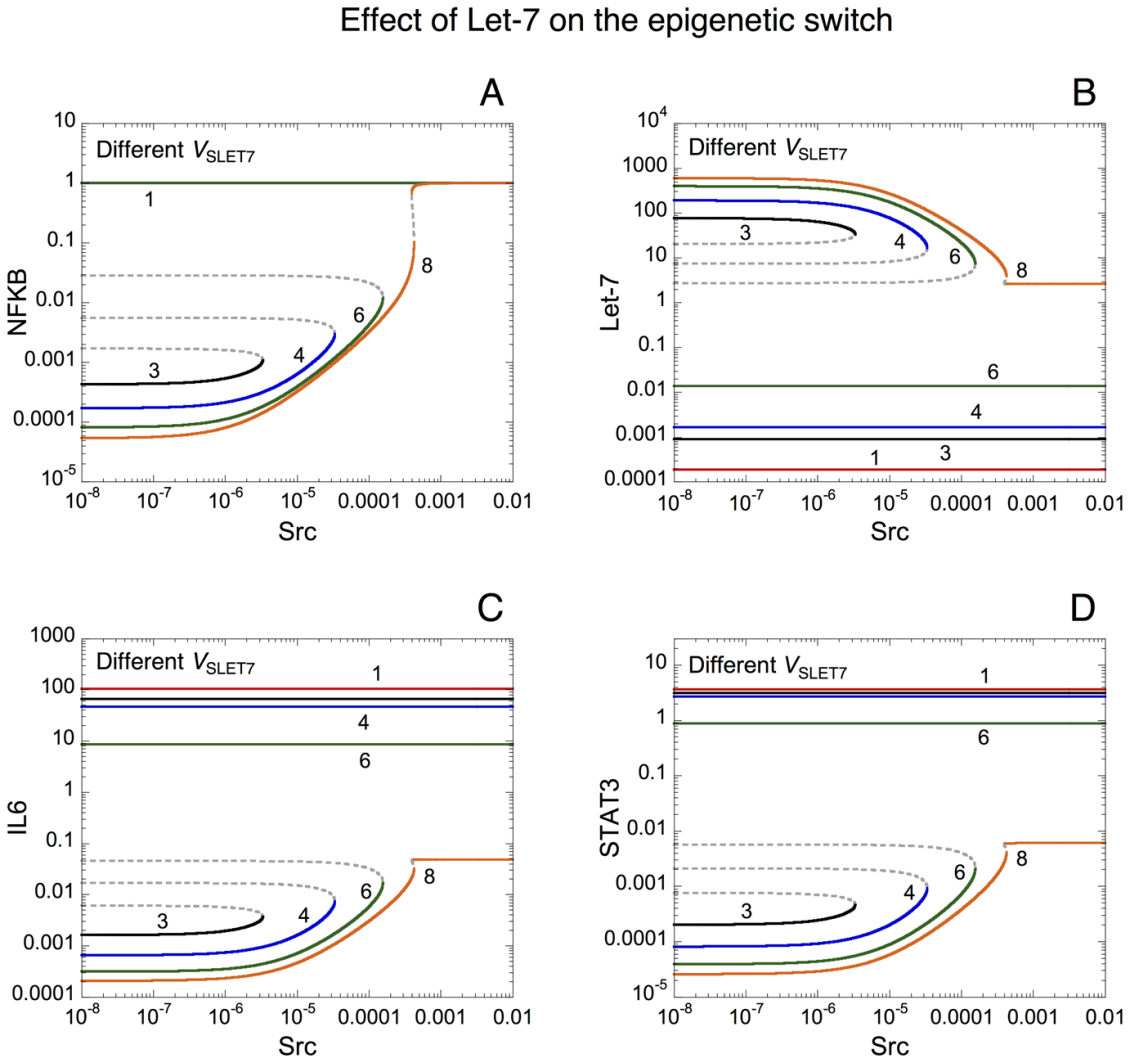
Effect of Let-7 microRNA on the dynamical behavior of the epigenetic switch leading to cell transformation. Bifurcation diagrams showing the steady-state levels of NF-κB, Let-7, IL6 and STAT3 as a function of the inflammatory signal, Src, are shown in panels A to D, respectively. For each case, different rates of synthesis of Let-7, *V*_SLET7_ are considered: *V*_SLET7_ is equal to 1, 3, 4, 6 and 8. Solid curves correspond to stable steady states, while dashed curves represent unstable states. A transformed state of the cell defined by high levels of NF-κB, IL6 and STAT3 together with a low level of Let-7 is present regardless of the level of Src when *V*_SLET7_ is small, *V*_SLET7_ = 1. For higher rates of synthesis of Let-7 (*V*_SLET7_ = 3, 4 and 6), the system is defined by an irreversible bistable switch characterized by the coexistence of low and high levels of the different variables for small values of Src, and by a low level of Let-7 and high levels of NF-κB, IL6 and STAT3 at high values of Src. An increase in *V*_SLET7_ from 3 to 6 moves the switch leading to an inflammatory response and cell transformation to higher levels of Src. When *V*_SLET7_ is elevated, *V*_SLET7_ = 8, the inflammatory response is very weak, regardless of the level of Src. Note that in the upper branch of the bistable switch, we cannot distinguish the different curves because their levels are very close to each other. Parameter values used in the simulations are given in Table 2.

Let-7 microRNA and PTEN are tumor suppressors and negative regulators of the epigenetic switch leading to cell transformation (see [7] as well as Fig. 1). By resorting to the computational model, we can assess the role of these tumor suppressors on the dynamics of the switch linking inflammation to cell transformation. To this end, we analyze the dynamical behavior of the model by means of bifurcation diagrams, which bring to light the steady-state levels of the system, i.e. equilibrium levels of the different variables of the model, as a function of a parameter value. In Fig. 3, the steady-state levels of NF-κB, Let-7, IL6 and STAT3 are shown as a function of the inflammatory signal, Src, for different rates of synthesis of Let-7, *V*_SLET7_. The model indicates that the epigenetic switch linking inflammation to cell transformation behaves as an irreversible bistable switch towards the inflammatory signal, Src. Indeed, in the presence of low levels of Src, the model predicts that a non-transformed state defined by low levels of NF-κB, IL6 and STAT3 together with a high level of Let-7 coexists with a transformed state characterized by high levels of NF-κB, IL6 and STAT3 together with a low level of Let-7. Depending on the value of initial conditions, the system reaches the non-transformed or the transformed state. In the presence of high level of Src, only the transformed state remains.

The model suggests that Let-7 microRNA acts as a tumor suppressor because it moves the threshold leading to cell transformation to higher level of Src (see Fig. 3A–D when *V*_SLET7_ increases from 3 to 8). The model also predicts that, when the rate of synthesis of Let-7 is too small, only the transformed state is present (see curves in Fig. 3 when *V*_SLET7_ = 1).

Steady-state levels of Let-7 and IL6 *vs* Src illustrated for different rates of transcription of PTEN, *V*_SMPTEN_, indicate that the tumor suppressor PTEN exhibits a similar effect as Let-7 on the dynamics of the epigenetic switch leading to cell transformation (see Fig. S2). Indeed, an increase in *V*_SMPTEN_ moves the threshold defining cell transformation to higher levels of Src.

### Effect of the oncogene Ras on the epigenetic switch leading to a transformed state

**Figure 4:**
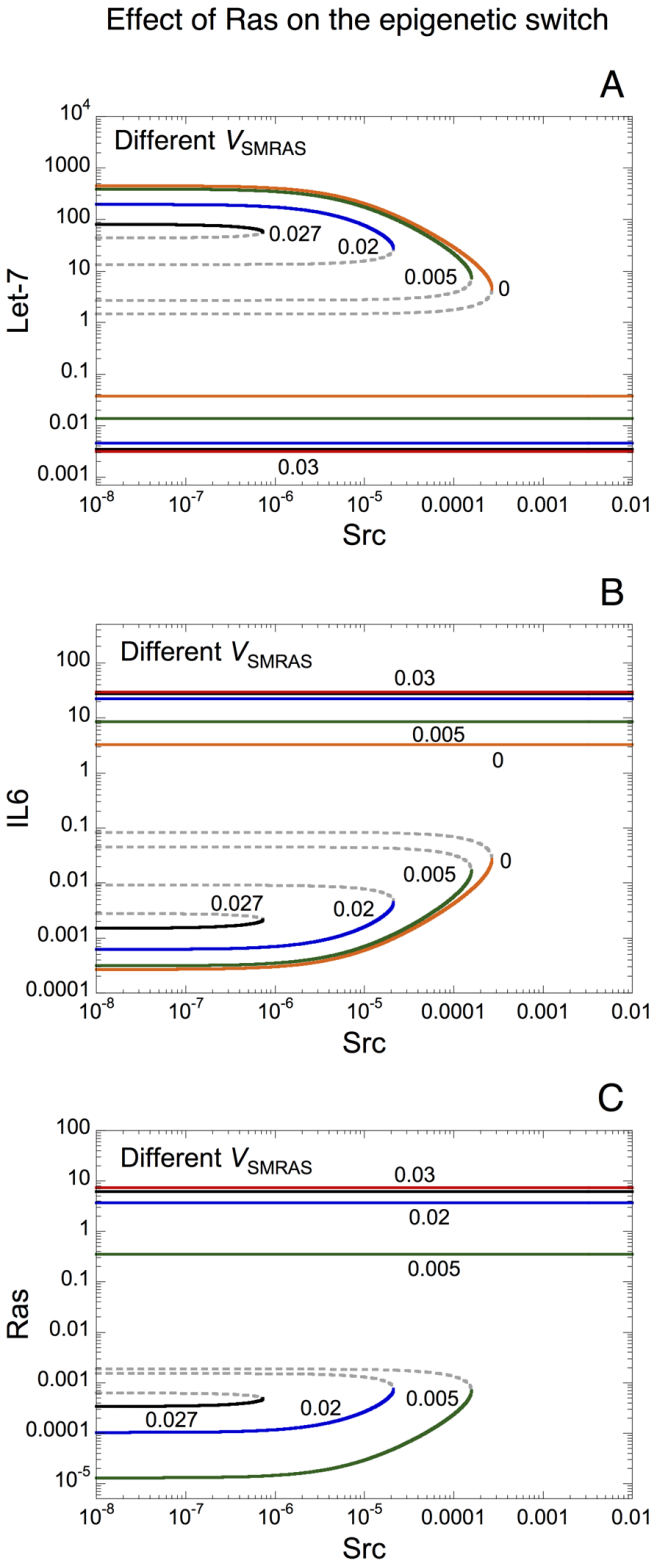
Effect of the oncogene Ras on the dynamical behavior of the epigenetic switch. Bifurcation diagrams showing the steady-state levels of Let-7, IL6 and Ras are shown respectively in panels A to C as a function of the inducer signal, Src. For each case, different rates of transcription of Ras, *V*_SMRAS_ are considered. *V*_SMRAS_ is equal to 0, 0.005, 0.02, 0.027 and 0.03. Solid curves: stable steady states; dashed curves: unstable states. A transformed state of the cell, defined by a low level of Let-7 and high levels of IL6 and Ras, is observed regardless of the level of Src when *V*_SMRAS_ is high (*V*_SMRAS_ = 0.03). For lower rates of transcription of Ras, the model predicts a bistable response as a function of Src. Starting from *V*_SMRAS_ = 0, an increase in *V*_SMRAS_ moves the threshold leading to cell transformation to smaller levels of the inflammatory signal, Src. Parameter values are as in Fig. 3, with *V*_SLET7_ = 6.

Contrary to Let-7 and PTEN, Ras is a positive regulator of the epigenetic switch leading to cell transformation (see Fig. 1 as well as [7], [43]). Bifurcation diagrams of Let-7, IL6 and Ras *vs* Src are shown for different rates of transcription of Ras, *V*_SMRAS_ in Fig. 4A–C, respectively. In the framework of the epigenetic switch linking inflammation to cancer, the model indicates that Ras acts as an oncogene by moving the threshold leading to cell transformation to smaller levels of the inflammatory signal, Src (see Fig. 4 when *V*_SMRAS_ increases from 0 to 0.027). For high level of Ras, i.e. *V*_SMRAS_ = 0.03, the model predicts that the cell is only present in a transformed state (low level of Let-7 together with high levels of IL6 and Ras) regardless of the level of Src.

### Cell transformation driven by stochastic transitions

**Figure 5:**
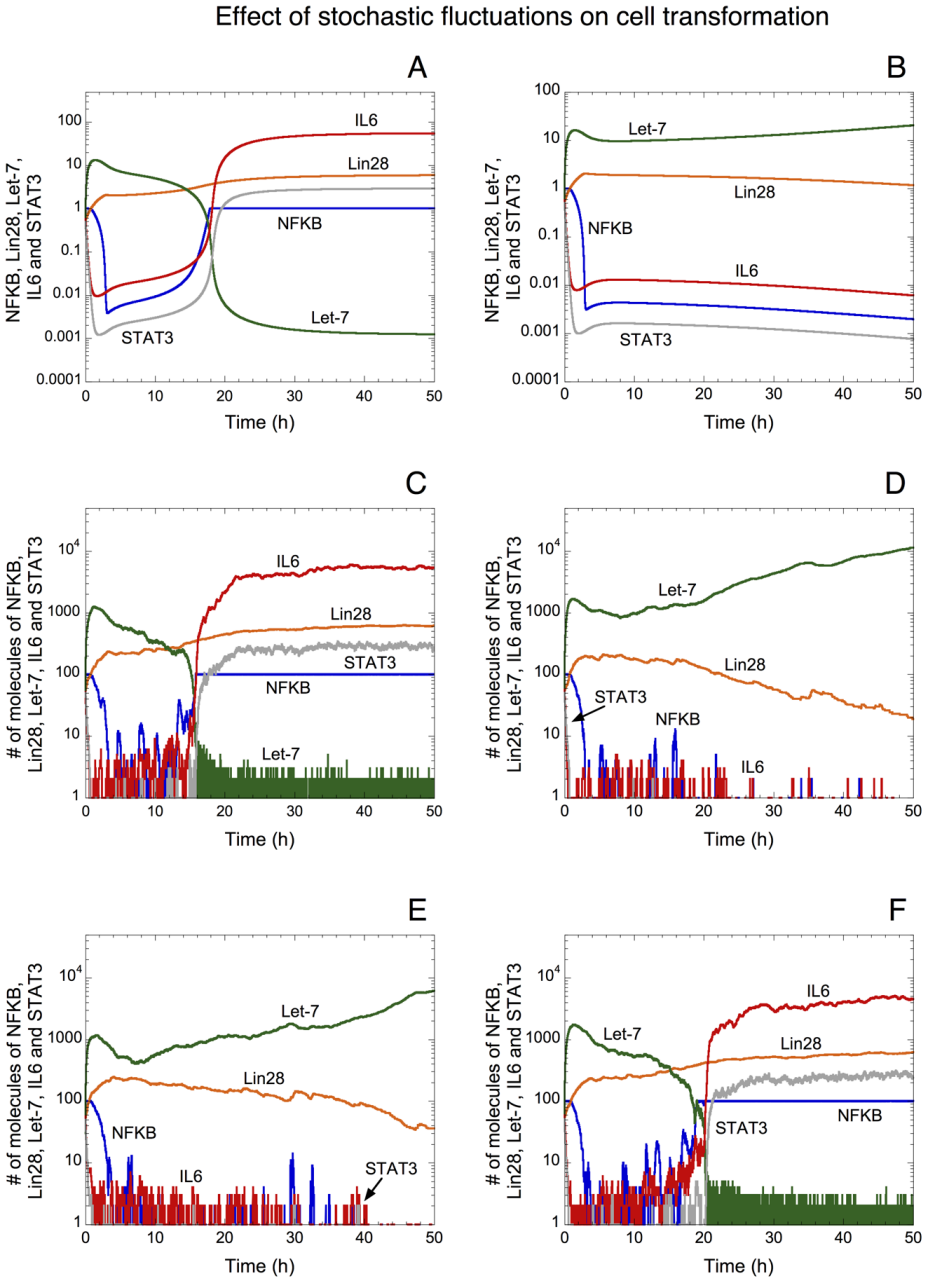
Effect of stochastic fluctuations on the epigenetic switch leading to cell transformation. Deterministic (A and B) *vs* stochastic time evolution (C–F) of NF-κB, Lin28, Let-7, IL6 and STAT3. For the same conditions, as a result of stochastic fluctuations, the switch leading to cell transformation may or may not occur (compare the deterministic simulations in A with the corresponding stochastic simulations in C and E, as well as the deterministic time course in B with the corresponding stochastic time evolution in D and F). Stochastic simulations were performed by means of the Gillespie's algorithm [63] using the stochastic version of the model for the epigenetic switch (see Table S1 in Supporting Information). The units on the axes for the stochastic curves are expressed in numbers of molecules. The corresponding concentrations for the deterministic trajectories are obtained by dividing the numbers of molecules by Ω expressed in units of 10^6^ L/N_A_, where N_A_ is Avogadro's number. Here, as well as in all stochastic simulations in this study, Ω = 100. Parameter values are as in Table 2 with Src = 0.000001 and *V*_SLET7_ = 3.5 in panels A, C, E; and with Src = 0.00001 and *V*_SLET7_ = 4 in panels B, D, F.

It has been shown in many biological systems that stochastic transitions may represent a driving force in development and in cell fate decisions [44]. Here, by resorting to a stochastic version of the model for the epigenetic switch linking inflammation to cancer (see Table S1 in Supporting Information), we assess the effect of stochastic fluctuations on the dynamics of the epigenetic switch. Deterministic (Fig. 5A) and the corresponding stochastic time evolution (Fig. 5C, E) of NF-κB, Lin28, Let-7, IL6 and STAT3 indicate that while deterministic transition to a transformed state of the cell occurs at about t = 20 h (Fig. 5A), stochastic fluctuations may prevent the occurrence of such transition (compare Fig. 5C and 5E). The opposite is also observed: stochastic fluctuations driving the occurrence of cell transformation while the deterministic version of the model predicts a non-transformed state (compare Fig. 5B with Fig. 5D and 5F).

Could the tumor suppressors, Let-7 microRNA and PTEN, or the oncogene Ras influence the occurrence of stochastic transitions in the model linking inflammation to cell transformation? To answer that question, we constructed deterministic as well as stochastic bifurcation diagrams of IL6 *vs* Src for different rates of synthesis of Let-7, PTEN, or Ras.

**Figure 6:**
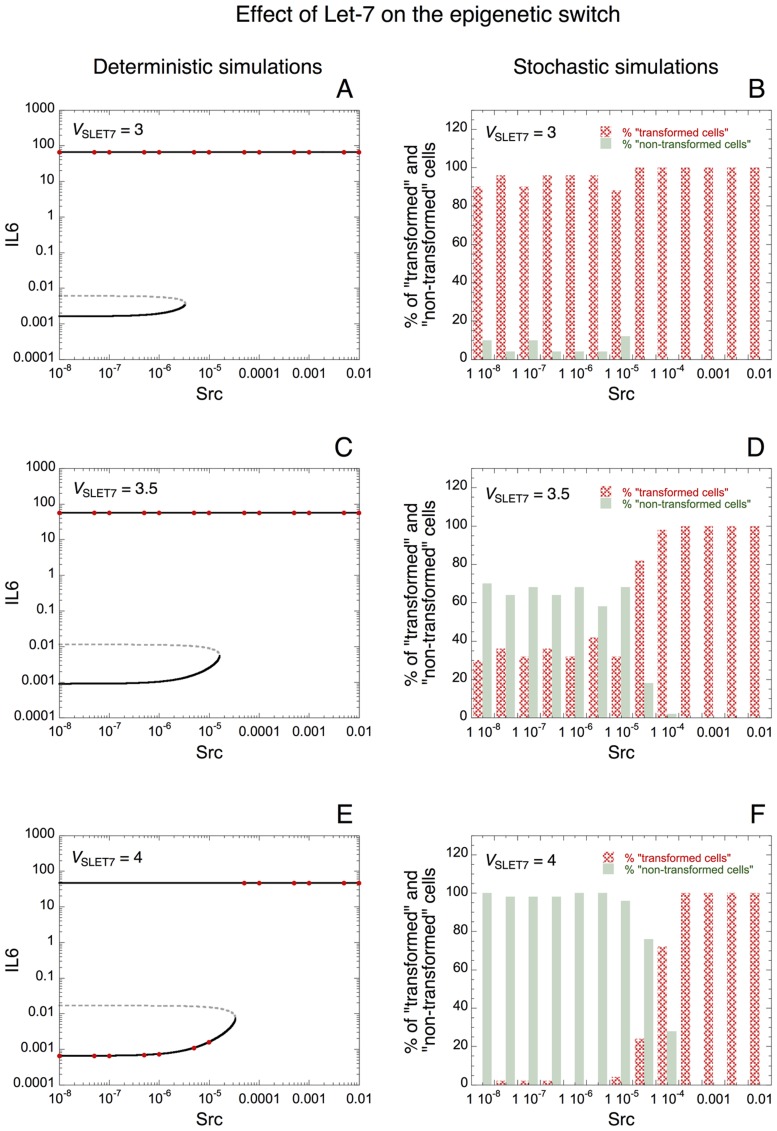
Effect of Let-7 microRNA on the robustness of the epigenetic switch towards molecular noise. (A, C, E) Deterministic simulations. The steady-state level of IL6 *vs* Src is represented for *V*_SLET7_ = 3 (A), 3.5 (C), and 4 (E). Red dots correspond to the steady state level of IL6 reached with the initial conditions used (see Methods). Solid curves: stable steady states; dashed curves: unstable states. Stochastic simulations showing the proportion of transformed and non-transformed state of the cells *vs* Src are represented in panels B, D and F for the conditions of panels A, C, E, respectively. Transformed state is defined by high level of IL6 together with low level of Let-7, while low level of IL6 together with high level of Let-7 characterizes a non-transformed state (see also stochastic time series in Fig. 5). The proportion of transformed *vs* non-transformed state of the cells is estimated with 50 stochastic cells for each condition, after a transient time of 50 h. For low levels of Src, nearly all cells are present in a transformed state when *V*_SLET7_ = 3 (B); a mixed population of transformed and non-transformed cells is present when *V*_SLET7_ = 3.5 (D); while nearly all cells are present in a non-transformed state when *V*_SLET7_ = 4 (F). Parameter values are as in Fig. 3.

Deterministic bifurcation diagrams of IL6 *vs* Src are shown when the rate of synthesis of Let-7, *V*_SLET7_ is equal to 3, 3.5 and 4 in Fig. 6A, C, E, respectively. The corresponding stochastic bifurcation diagrams are illustrated in Fig. 6B, D, F, respectively. With the initial conditions used (see section: “Methods”), deterministic simulations indicate that the cell is always in a transformed state when *V*_SLET7_ = 3 or 3.5 (red dots in Fig. 6A, C); while, when *V*_SLET7_ = 4, the cell is in a non-transformed state for low levels of Src and in a transformed state for higher levels of Src (see red dots in Fig. 6E). The corresponding stochastic simulations for the same conditions indicate that, for low levels of Src, the proportion of cells present in a non-transformed state is larger when *V*_SLET7_ increases (compare Fig. 6B, D and F). Thus, the model indicates that high levels of Let-7 may enhance the robustness of the non-transformed state to stochastic fluctuations by reducing the occurrence of stochastic switches leading to cell transformation. The fact that the deterministic unstable steady state is closer to the stable steady state with a low level of IL6 (non-transformed state) when *V*_SLET7_ is small is already an indication that the latter stable steady state may be less robust to stochastic fluctuations in the presence of low levels of Let-7 (compare dashed curves in Fig. 6A, C, E).

Similarly to Let-7 microRNA, the model shows that the tumor suppressor PTEN is also able to increase to robustness of the cell towards stochastic cell transformation (Fig. S3). Deterministic bifurcation diagrams of IL6 steady-state levels *vs* Src indicate that the stable steady state corresponding to a non-transformed state (low level of IL6) is larger when the rate of transcription of PTEN, *V*_SMPTEN_ is high (compare Fig. S3A, C). In the stochastic approach, the model shows that increasing the level of PTEN reduces the occurrence of cell transformation for low level of Src (compare Fig. S3B and S3D).

**Figure 7:**
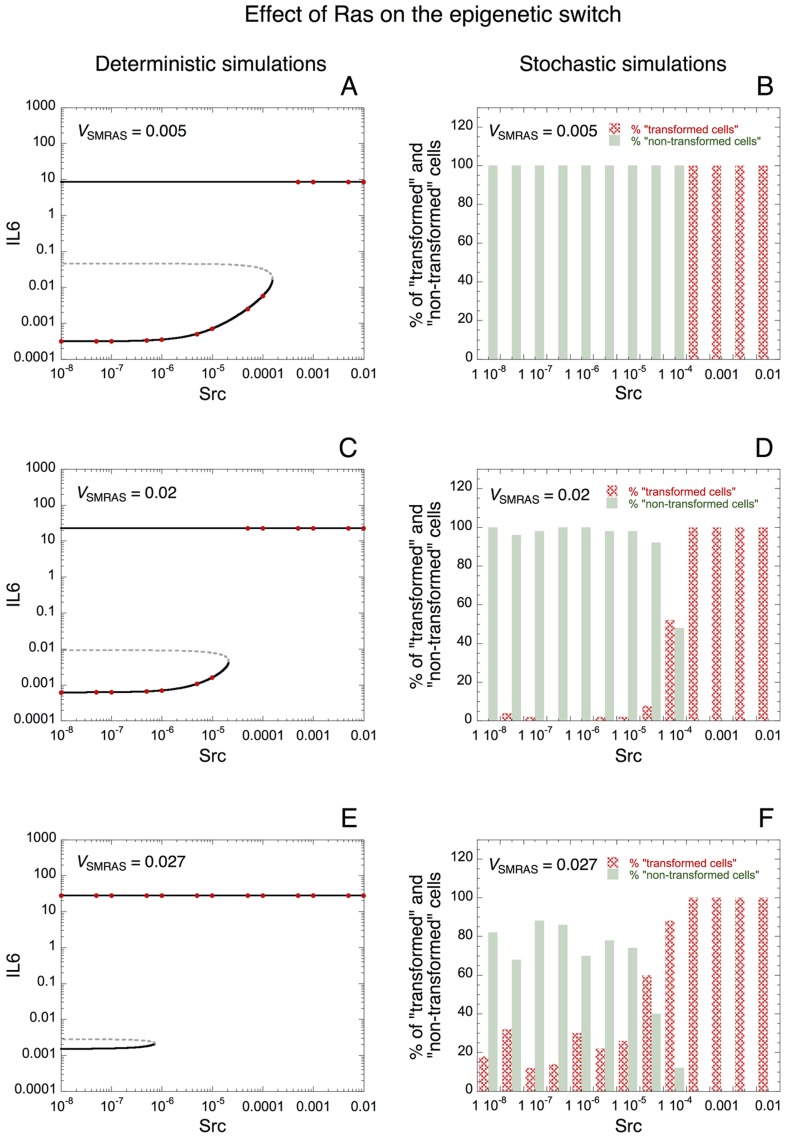
Effect of Ras oncogene on the robustness of the switch towards stochastic fluctuations. (A, C, E) Deterministic simulations. The steady-state level of IL6 *vs* Src is represented for *V*_SMRAS_ = 0.005 (A), 0.02 (C), and 0.027 (E). Red dots correspond to the steady state level of IL6 reached with the initial conditions considered (see Methods). Solid curves: stable steady states; dashed curves: unstable states. Stochastic simulations showing the proportion of transformed and non-transformed cells *vs* Src for the conditions of panels A, C, E are shown in panels B, D and F, respectively. For low levels of Src, the robustness of the non-transformed state decreases when the rate of synthesis of the oncogene Ras, *V*_SMRAS_, increases (compare panels B, D and F). Indeed, even with a low level of Src, the proportion of transformed cells increases when *V*_SMRAS_ is high. Parameter values are as in Fig. 3 with *V*_SLET7_ = 6.

Deterministic and stochastic simulations performed for different rates of transcription of the oncogene Ras, *V*_SMRAS_ suggest that, contrary to Let-7 or PTEN, an elevated level of Ras increases the sensitivity of the switch to stochastic fluctuations for the occurrence of cell transformation (see Fig. 7). Indeed, deterministic bifurcation diagrams indicate that the stable steady state with a low level of IL6 (corresponding to a non-transformed state) gets smaller when *V*_SMRAS_ increases from 0.005 to 0.027 (see Fig. 7A, C, E). The corresponding stochastic simulations suggest that a robust switch without transformed cells, for low level of Src, is present when *V*_SMRAS_ is small (Fig. 7B). By increasing the level of Ras, the switch leading to cell transformation becomes less robust to stochastic fluctuations and a significant proportion of transformed cells appear even for a low level of inflammatory signal, Src (Fig. 7D, F).

Besides ‘intrinsic noise’ coming from molecular noise (stochastic fluctuations), the dynamics of the inflammatory network may be also influenced by ‘extrinsic noise’ originating from to cell-to-cell variability in a population. In that framework, recent studies have shown that the dynamics of activation of NF-κB is heterogeneous within a cell population [40], [45], [46]. Those studies suggested that the dynamics of cell responding to inflammatory signaling is partly driven by stochastic processes, but seems mostly controlled by pre-existing variation in internal variables of the cells (extrinsic noise) [40], [45], [46].

To address the issue of cell-to-cell heterogeneity on the dynamics of the network, we will study, in the next section, the dynamics of the epigenetic switch in a heterogeneous cell population.

### Transformed versus non-transformed state of the cells in a heterogeneous cell population

In order to see if this epigenetic switch is relevant for human cancers, Iliopoulos and coworkers examined the expression levels of IL6 and Let-7 in cancer and normal breast, prostate, hepatocellular, and lung tissues [7]. They showed that cancer tissues have higher levels of IL6 and lower levels of Let-7 as compared to normal tissues. A negative correlation is also observed in the expression levels of Let-7 and IL6 for breast, prostate, and hepatocellular tissues [7].

**Figure 8:**
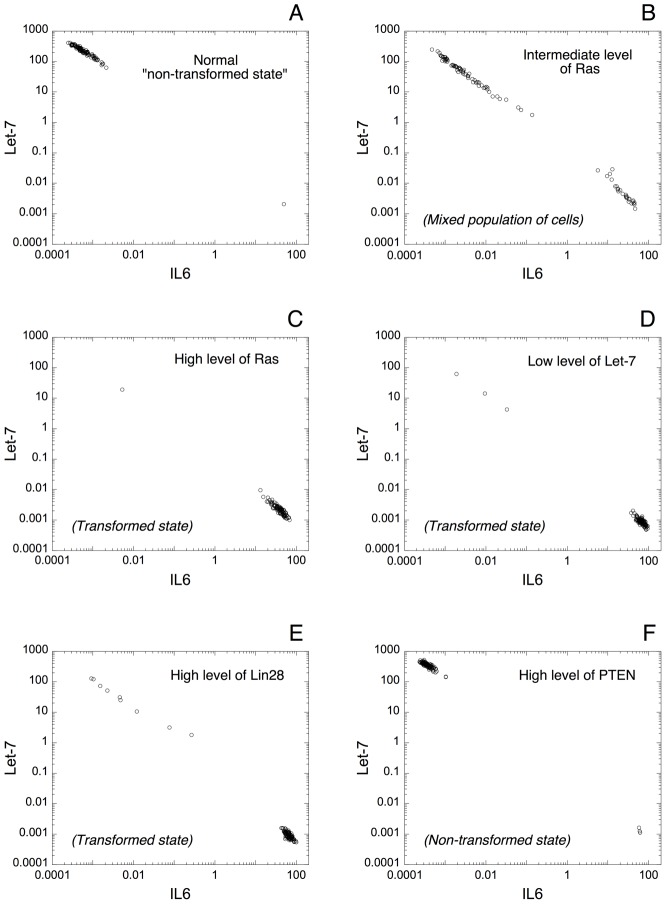
Expression of Let-7 *vs* IL6 in a heterogeneous cell population. Deterministic simulations are performed with 100 cells with 10% of random variation from the default value on each parameter of the model. Each dot corresponds to the state of one cell after 50 h of transient time. (A) Normal, non-transformed state of the cell population. Nearly all cells exhibit high levels of Let-7 and low levels of IL6. Parameter values are as in Fig. 3 with *V*_SLET7_ = 6. (B) From the condition in A, an increase in *V*_SMRAS_ from 0.005 to 0.02 generates a mixed population of transformed and non-transformed cells. (C) A further increase in *V*_SMRAS_ to 0.05 allows switching to a population of transformed cells (high levels of IL6 together with low levels of Let-7). From the condition in A, the switch of the cell population from a non-transformed to a transformed state can be also achieved by decreasing the rate of synthesis of Let-7, *V*_SLET7_ from 6 to 3 (D), or by increasing the rate of synthesis of the transcription factor Lin28, *V*_SLIN28_ from 0.012 to 0.024 (E). (F) From the condition in C, an increase in the transcription rate of PTEN, *V*_SMPTEN_ from 0 to 0.5 allows recovering a normal, non-transformed state of the cell population. In each case, the model exhibits a negative correlation between Let-7 and IL6 expression, as observed in the experiments [7]. Other default values of the parameters are as in Table 2 with Src = 0.000001.

By resorting to the deterministic model proposed for the switch leading to cell transformation, we are able to reproduce qualitatively these experimental observations. To do so, we analyze the expression pattern of IL6 and Let-7 in a model for a heterogeneous cell population. For each cell in the population, which counts 100 cells, all the parameters values are chosen randomly with 10% of variation from their default value (see Fig. 8).

The model for the cell population shows that a normal, non-transformed state can be achieved with high levels of Let-7 together with low levels of IL6 (Fig. 8A). From that state, the model predicts that an increase in the level of the oncogene Ras triggers cell transformation in a significant proportion of cells in the population (see Fig. 8B), which results in a mixed population of non-transformed and transformed cells. A further increase in Ras almost completely switches the cell population to a transformed state, defined by high levels of IL6 and low levels of Let-7 (Fig. 8C). The model indicates that by starting from a non-transformed state of the cell population (Fig. 8A), the switch of the cell population to a transformed state can be also triggered by reducing the rate of synthesis of Let-7, *V*_SLET7_ (Fig. 8D), or by increasing the level of Lin28 (Fig. 8E). Finally, the model predicts that a transformed state of the cell population (Fig. 8C) could switch back to a non-transformed state by increasing the level of the tumor suppressor PTEN (Fig. 8F). This latter result supports the experimental observations showing that elevated levels of PTEN induce a tumor-suppressive metabolic state [47].

In all cases, the model indicates, as in the experiments [7], that the expression pattern of Let-7 and IL6 is negatively correlated.

To assess the robustness of the model with respect to random variation of parameters in a heterogeneous cell population, we further illustrate the expression levels of Let-7 and IL6 in a heterogeneous cell population for increasing levels of random variation on parameters: 5%, 10%, 25% and 50% (see Fig. S4). Simulations show that even with large random variation on every parameter of the model (25% or 50%), most cells in the population are characterized by a non-transformed state in normal conditions (see Fig. S4G, S4J) as well as in the presence of high levels of both Ras and PTEN (Fig. S4I, S4L). In contrast, in each case, most cells in the population are defined by a transformed state in the presence of high levels of Ras (Fig. S4H, S4K). This result indicates that the model is quite robust to random variations on parameter values.

By resorting to the deterministic model for a heterogeneous cell population, we are also able to reproduce the dynamical behavior of NF-κB activation. Indeed, single-cell analysis revealed that the activation of NF-κB is heterogeneous and is a digital on-off process with fewer cells responding at lower doses of inflammatory signal [45], [46]. The model accounts for this observation by showing that the expression levels of Let-7 and NF-κB are characterized by two main states: (1) high levels of Let-7 and low levels of NF-κB, or (2) low levels of Let-7 and high levels of NF-κB (see Fig. S5). The number of responding cells, defined by high levels of NF-κB and low levels of Let-7 increases by increasing the level of inflammatory signal, Src (compare panels A to C in Fig. S5).

### miR-21 may trigger cell transformation

The microRNA miR-21 is overexpressed and promotes invasion in pancreatic ductal adenocarcinoma [48]. Moreover, it was shown experimentally that miR-21 is an activator in the regulatory feedback loop linking inflammation to cell transformation [20]. Indeed, STAT3 promotes the expression of miR-21, which results in the down-regulation of the tumor suppressor PTEN leading to an activation of NF-κB (see Fig. 1). A transient expression of miR-21 can induce the epigenetic switch, which leads to cell transformation and formation of mammospheres in mouse xenografts [20]. The positive feedback loop between NF-κB, Lin28, Let-7, IL6 is crucial for this process of cellular transformation because the concomitant overexpression of miR-21 together with the inhibition of Lin28 considerably reduce cell transformation and mammospheres formation [20].

**Figure 9:**
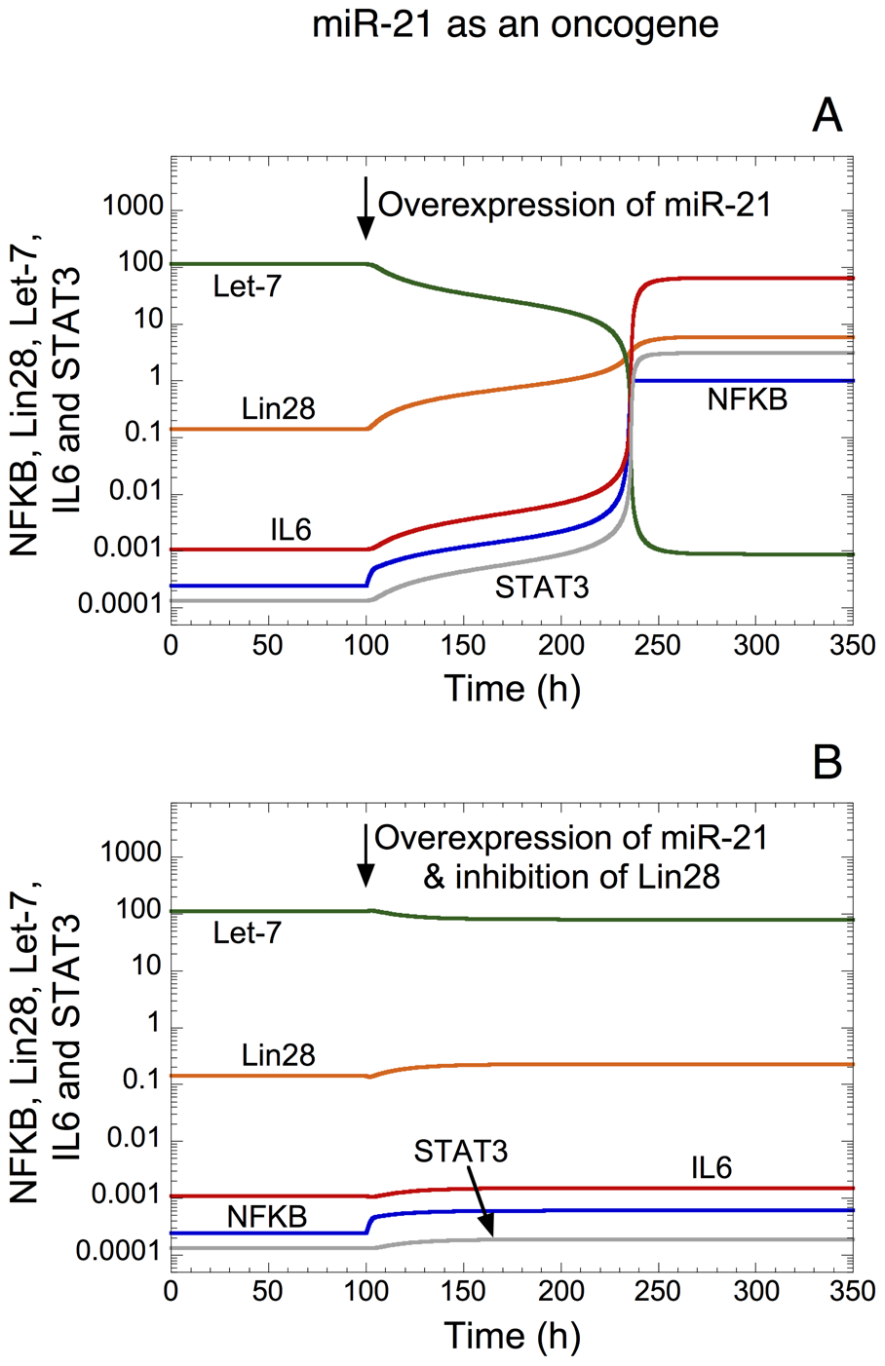
miR-21 microRNA acts as an oncogene promoting cell transformation. Time evolution of NF-κB, Lin28, Let-7, IL6 and STAT3 is shown in the presence of an overexpression of miR-21 (panel A for t>100 h), and in the presence of an overexpression of miR-21 together with an inhibition of Lin28 (panel B for t>100 h). For t<100 h, normal condition, non-transformed state of the cell characterized by a high level of Let-7 and low levels of Lin28, NF-κB, IL6 and STAT3. (A) The overexpression of miR-21, *V*_SMIR21_ = 4000, triggers cell transformation. (B) The same overexpression of miR-21 does not trigger cell transformation if Lin28 is inhibited, *V*_SLIN28_ = 0.008 instead of 0.012. Other parameter values are as in Fig. 3 with *V*_SLET7_ = 3, *V*_SMPTEN_ = 0.1, *Src* = 0.00001.

In the model, from a non-transformed state of the cell characterized by a high level of Let-7 and low levels of NF-κB, Lin28, IL6, and STAT3, the overexpression of miR-21 triggers the switch to cell transformation, which results in a low level of Let-7 and high levels of NF-κB, Lin28, IL6, and STAT3 (see Fig. 9A). The model qualitatively reproduces the experimental observation by showing that the concomitant overexpression of miR-21 together with a partial inhibition of Lin28 is not able to trigger the switch to cell transformation (Fig. 9B). Indeed, only a small increase in the level of NF-κB, Lin28, IL6, and STAT3 and a small decrease in Let-7 are observed (see Fig. 9B for t>100 h).

Iliopoulos and coworkers also showed, in colon adenocarcinomas, a positive correlation in the expression pattern of miR-21 and STAT3 as well as a negative correlation in the expression pattern of miR-21 and PTEN [20]. By resorting to the deterministic model for a heterogeneous cell population, we can reproduce qualitatively these expression patterns (see Fig. S6). A low rate of synthesis of miR-21 generates a mixed population of non-transformed and transformed cells (Fig. S6A, B). From that condition, an increase in the rate of synthesis of miR-21 switches the cell population to a transformed state characterized by high levels of miR-21 and STAT3 together with low levels of PTEN (Fig. S6C, D). From the latter condition, a reduction in the level of Lin28 brings back a large proportion of cells to a non-transformed state defined by low levels of miR-21 and STAT3 together with high levels of PTEN (Fig. S6E, F). This result supports the experimental observations showing the reduced number of mammospheres formation in the presence of Lin28 inhibition [20].

### Control of cell transformation by competing endogenous RNA, ceRNA

Recent hypothesis suggests that some messenger RNAs, competing endogenous RNAs (ceRNA), could possess a regulatory role, independently of their protein-coding function, by their ability to compete for microRNA binding [26], [27], [29]. ceRNA could act as natural microRNA sponge [49]. In that context, by sharing the same microRNAs, it was shown that the expression of the tumor suppressor PTEN and its pseudogene PTENP1 are positively correlated. Indeed, even if the pseudogene PTENP1 does not encode a functional protein, PTENP1 mRNA may regulate the expression of the tumor suppressor PTEN by competing for their common microRNA [28]. It was also shown that the pseudogene PTENP1 is mutated in some cancers [28].

**Figure 10:**
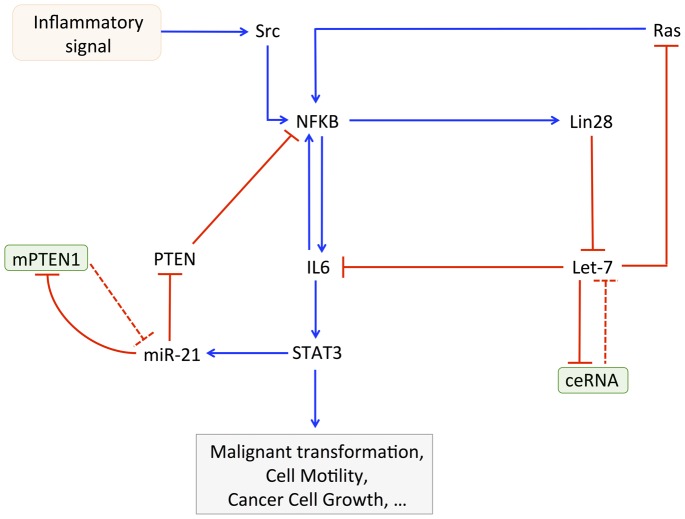
Wiring diagram of the model for the epigenetic switch linking inflammation to cell transformation, including two competing endogenous RNAs (ceRNAs) (see also Fig. 1). A hypothetical ceRNA can form an inhibitory complex with Let-7 microRNA. Moreover, the addition of the messenger RNA of PTEN1, mPTEN1, which can sponge the microRNA miR-21, allows us to assess the effect of the pseudogene of PTEN (mPTEN1) on the epigenetic dynamics of cell transformation.

By resorting to our computational model linking inflammation to cell transformation, we will analyze here the regulatory role of a generic ceRNA, which could compete for the binding to Let-7 microRNA as well as the role of the pseudogene PTEN1, which competes with PTEN mRNA for the binding to miR-21 (see Methods for more details as well as the wiring diagram in Fig. 10).

Bifurcation analyzes of the steady-state levels of NF-κB, Lin28, Let-7, IL6, STAT3, and ceRNA *vs* Src for different rates of synthesis of ceRNA, *V*_SCERNA_, suggest that a competing-endogenous RNA for the binding to Let-7 microRNA could act as an oncogene by promoting the switch to cell transformation (see Fig. S7). Indeed, the model predicts that an increase in *V*_SCERNA_ from 0 to 1 moves the switch from a non-transformed to a transformed state to smaller levels of the inflammatory signal, Src. Furthermore, the model also shows that if the level of ceRNA is too high, i.e. *V*_SCERNA_ = 2, the cell is always present in a transformed state defined by high levels of NF-κB, Lin28, IL6, STAT3, and ceRNA together with a low level of Let-7.

By means of stochastic simulations, the model indicates that an elevated level of ceRNA decreases the robustness of the non-transformed state of the cell towards stochastic fluctuations (see Fig. S8). Indeed, by increasing *V*_SCERNA_, the proportion of transformed cells at low levels of Src is larger (compare Fig. S8B, D, F).

**Figure 11:**
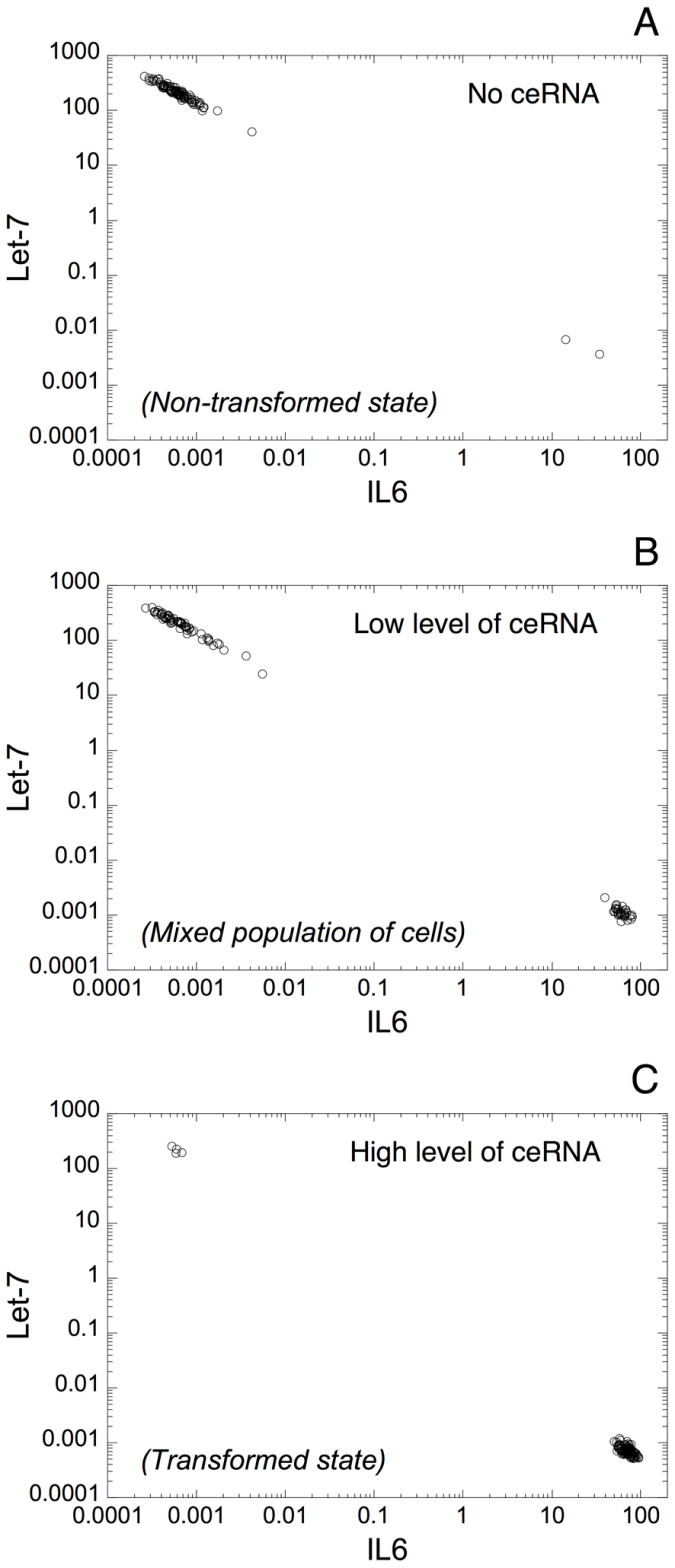
Effect of ceRNA binding to Let-7 microRNA on the dynamics of a heterogeneous cell population. Levels of expression of Let-7 *vs* IL6 are illustrated in a cell population without ceRNA, *V*_SCERNA_ = 0 in A; with a low level of ceRNA, *V*_SCERNA_ = 0.1 in B; and with a higher level of ceRNA, *V*_SCERNA_ = 0.2 in C. 100 cells are considered in the simulations with 10% of random variation from the default value on all parameters. (A) In the absence of ceRNA, the cell population is mainly in a non-transformed state characterized by high levels of Let-7 and low levels of IL6. (B) A low level of ceRNA generates a mix population of transformed and non-transformed cells. (C) In the presence of a higher level of ceRNA, nearly all cells switched to a transformed state (low levels of Let-7 together with high levels of IL6). Default parameter values are as in Table 2 with *V*_SLET7_ = 6 and Src = 0.000001. Let-7 and IL6 levels are calculated after 50 h of transient.

The deterministic model for a heterogeneous cell population shows that, in the absence of ceRNA, the expression levels of Let-7 and IL6 are negatively correlated with high levels of Let-7 together with low levels of IL6, which corresponds to a non-transformed state of the cell population (Fig. 11A). An increase in ceRNA progressively promotes the switch of the cell population to a transformed state characterized by low levels of Let-7 together with high levels of IL6 (see Fig. 11B, C).

Thus, the model predicts that the effect of a ceRNA for Let-7 binding is very similar to the effect of Ras oncogene on the dynamics of the epigenetic switch leading to cell transformation.

Finally, the model indicates that high levels of PTEN1 mRNA delay or eventually suppress the occurrence of cell transformation (see Fig. S9 where the time evolution of NF-κB, IL6, miR-21, and PTEN is represented for different rates of transcription of the pseudogene PTEN1, *V*_SMPTEN1_). This supports the experimental observations showing that PTEN ceRNAs exhibit tumor-suppressive properties [50].

## Discussion

Rudolf Virchow made the first connection between chronic inflammation and cancer in 1863 [5]. Nowadays, molecular links between inflammation and oncogenic transformation have been established [6]. However, until recently, the molecular pathways linking inflammation to cellular transformation were unknown [7]. In the latter study, the authors showed that ER-Src oncoprotein by treatment with tamoxifen could convert a non-transformed cell line to a transformed state within 24–36 h. They also demonstrated that this cell transformation is mediated by an inflammatory positive feedback loop driven by NF-κB, Lin28, Let-7, and IL6 (see [7] and Fig. 1).

Based on these experiments [7], [20], we proposed here a computational model to account for the dynamics of the positive inflammatory feedback loop leading to cell transformation. The model includes the regulations between NF-κB, Lin28, Let-7, IL6, Ras, STAT3, miR-21, and PTEN (see Fig. 1).

First the model accounts for the experimental observations showing that the non-transformed state, characterized by low levels of activators of the switch (NF-κB, Lin28, IL6, and STAT3) together with high levels of inhibitors of the switch (Let-7 and PTEN), is stable without the inflammatory signal, Src (Fig. 2). As in the experiments, a transient activation of Src for only 5 min triggers cell transformation within 60 h, while a constant level of Src may elicit the switch to a transformed state within 35 h (see Fig. 2). The model also accounts for the fact that maintenance of the transformed state needs the presence of the positive feedback loop between NF-κB, Lin28, Let-7, and IL6. Indeed, a transient inhibition of NF-κB, Lin28, or IL6 abolishes the transformed state of the cell (Fig. S1).

In the model, each component is embedded at least in one positive feedback (PF) loop (see wiring diagram in Fig. 1). The main PF loop is based on the regulations between NF-κB, Lin28, Let-7 and IL6. The second PF loop rests on the interactions between NF-κB, Lin28, Let-7 and Ras. The third PF loop is the mutual activation between NF-κB and IL6, while the fourth PF loop is driven by the regulatory interactions between NF-κB, IL6, STAT3, miR-21, and PTEN. Each component in the network can be clustered into one of the following two groups: activators (oncogenes such as NF-κB, Lin28, Ras, IL6, STAT3 and miR-21) or inhibitors of the switch leading to cell transformation (tumor suppressors such as Let-7 and PTEN).

By resorting to bifurcation analyzes, we show that an irreversible bistable switch between a transformed and a non-transformed state is at the core of the dynamics of the epigenetic switch linking inflammation to cancer (see Figs. 3, 4, S2). The model suggests that Let-7 and PTEN act as tumor suppressors by increasing the threshold of inflammatory signal, Src, at which the switch to cell transformation occurs (see Figs. 3 and S2, respectively). On the opposite, the model shows that Ras exhibits oncogenic properties by reducing the threshold of Src at which cell transformation happens (see Fig. 4).

Thus, at any moment, there is a sensitive balance in the relative levels of oncogenes and tumor suppressors that defines the state of the cell: non-transformed versus transformed.

The importance of stochastic gene expression and stochastic transitions has been highlighted is many different biological contexts [51], [52]. A study showed that a reduction of stochastic transitions could enhance cellular memory [53]. Stochastic mechanisms have been involved in the differentiation of mature subsets of T lymphocytes [54]. Stochastic gene fluctuations could drive the phenotype diversity in HIV-1 [55], and stochastic epigenetic variation has been proposed to be a major force of development, evolutionary adaptation and disease [44]. Stochastic phenomena could also play an important role in cancer. Indeed, stochastic appearance of mammary tumors has been observed experimentally [56]. A model showed that the stochastic effect due to the finite size of active stem cell population could greatly influence the dynamics of cancer evolution [57]. Stochastic fluctuations may also control nonheritable cell variability, which could drive the evolutionary rate of cancer progression [58]. Finally, it was shown both theoretically and experimentally that stochastic state transitions generate phenotypic equilibrium in populations of cancer cells [59].

Here, we show that stochastic fluctuations may be responsible for transitions between a non-transformed and a transformed state of the cell (Fig. 5). Furthermore, the model predicts that the tumor suppressors Let-7 and PTEN increase the robustness of the non-transformed state of the cell towards stochastic fluctuations (Figs. 6 and S3, respectively), while the oncogene Ras decreases this robustness (Fig. 7). Thus, besides the crucial role of multiple and successive genetic mutations in the process of cell transformation and cancer development [1], the model points out, based on experimental observations [7], [20], the important effect of epigenetic mechanisms and stochastic transitions on the dynamics of cell transformation.

The positive inflammatory feedback loop described here seems relevant in human cancers. Indeed, cancer tissues have lower levels of Let-7 and higher levels of IL6 as compared to normal tissues, and an inverse relationship is also observed in the expression pattern of Let-7 and IL6 in prostate, breast, and hepatocellular tissues [7]. Moreover, it was shown that a related microRNA inflammatory feedback loop controls hepatocellular oncogenesis [60]. Here, deterministic simulations of the model in a heterogeneous cell population allow accounting qualitatively for these observations by showing this negative correlation in the expression of Let-7 and IL6 (Fig. 8). The model exhibits high levels of Let-7 and low levels of IL6 in normal conditions (Fig. 8A). As suggested by the model, an increase in the level of the oncogene Ras, in the level of Lin28 or a decrease in Let-7 triggers the switch of the cell population to a transformed state (Fig. 8B–E); while, from a transformed state, an increase in the tumor suppressor PTEN allows recovering a normal, non-transformed, state of the cell population (Fig. 8F).

Here, we consider separately the two kinds of noises that can emerge in such dynamical system: ‘intrinsic’, due to molecular noise, and ‘extrinsic’, originating from cell-to-cell variability. Of course, in real, physiological conditions, these two kinds of noises combine within a cell population. Thus, a combination of both noises (intrinsic and extrinsic) may be a driving force to trigger, in a random manner, the occurrence of the switch leading to a transformed state of the cell. The latter source of noise resulting from cell-to-cell variability seems to have an important role leading to a heterogeneous activation of NF-κB under inflammatory signaling [45].

The model also accounts for the fact that miR-21 can be viewed as an oncogene. Moreover, the positive inflammatory feedback loop between NF-κB, Lin28, Let-7, and IL6 is important for the oncogenic property of miR-21 [20]. Indeed, we show, as in the experiments, that an overexpression of miR-21 elicits cell transformation, while a concomitant overexpression of miR-21 together with an inhibition of Lin28 greatly impede this transformation (Figs. 9 and S6).

It was recently hypothesized that messenger RNAs, transcribed pseudogenes, and long non-coding RNAs may interact with each other using microRNA response element, MREs [27]. It is suggested that those competing endogenous RNAs (ceRNAs) form a large regulatory network, which could play important roles in normal and pathological conditions, such as cancer or cell differentiation [27], [61], [62].

By using our computational model for the epigenetic switch linking inflammation to cancer, we analyzed the dynamical consequences on cell transformation of the addition of a ceRNA competing for the binding to Let-7 microRNA as well as the addition of the transcribed pseudogene PTEN1 (see wiring diagram in Fig. 10). The model shows that increasing the level of a ceRNA competing for Let-7 binding reduces the threshold of Src at which the switch to cell transformation occurs (Fig. S7). In the presence of ceRNA, the non-transformed state of the cell is also less robust towards stochastic fluctuations (Fig. S8). The dynamics of a heterogeneous cell population predicts that an elevated level of ceRNA leads to the switch of the cell population to a transformed state (Fig. 11). Thus, the model suggests that a ceRNA competing for Let-7 binding will sponge the available Let-7 microRNA, which promotes cell transformation. Such ceRNA may thus be viewed as an oncogene.

On the opposite, the model suggests that the addition of a ceRNA such as PTEN1 mRNA, competing with PTEN mRNA for binding to miR-21 microRNA, could act as a tumor suppressor inhibiting the occurrence of cell transformation (Fig. S9). This result holds with experimental observations showing the potential role of PTEN1 mRNA, or of other ceRNAs for PTEN, as tumor suppressors [26], [28], [50].

In summary, the model proposed here brings to light a comprehensive qualitative picture of the dynamics of the epigenetic switch linking inflammation to cell transformation and cancer. The model predicts that bistability is at the core of the underlying mechanism driving the switch between a non-transformed and a transformed state of the cell. Activators of the switch (oncogenes) and inhibitors of the switch (tumor suppressors) regulate the occurrence of cell transformation by modulating the threshold of inflammatory signal (Src) at which the switch occurs. The model also suggests that stochastic fluctuations could be a driving force for cell transformation, and predicts that tumors suppressors and oncogenes render the non-transformed state of the cell respectively more or less robust towards stochastic fluctuations. Finally, depending on their microRNA targets, the model shows that ceRNAs may act as oncogenes or as tumor suppressors, which points out the potential role of ceRNAs in the regulation of cell transformation.

## Methods

The model is described by a set of 14 kinetic equations (see first subsection below: “Kinetic equations of the model”) representing the time evolution of the concentration of the main variables driving the dynamics of the epigenetic switch linking inflammation to cell transformation. The different variables of the model are defined in Table 1, while the description of the parameters, together with their numerical values used in the simulations, are found in Table 2. The stochastic version of the model is presented in Table S1 (see Supporting Information). It consists of a set of reactions, which are directly related to the deterministic kinetic reactions and is simulated with the Gillespie algorithm [63].

**Table 2:** Parameters of the model.

Symbol	Definition	Numerical value
*k*_AA1NFKB_	Rate constant for the activation of NFKB by Src	10
*k*_AA2NFKB_	Rate constant for the activation of NFKB by IL6	0.09
*k*_AA3NFKB_	Rate constant for the activation of NFKB by Ras	1
*K*_IPTEN_	Michaelis constant for the inhibition of NFKB activation by PTEN	5
*V*_DNFKB_	Maximum rate for NFKB inactivation	0.01
*Src*	Src kinase oncoprotein (signal triggering an inflammatory response leading to an activation of the epigenetic switch)	
*K*_ANFKB_	Michaelis constant for the activation of NFKB	0.01
*K*_INFKB_	Michaelis constant for the inhibition of NFKB	0.02
*NFKB*_T_	Total concentration of NFKB	1
*V*_SLIN28_	Maximum rate of synthesis of Lin28	0.012
*K*_A1NF_	Michaelis constant for the activation of Lin28 synthesis by NFKB	0.01
*k*_DLIN28_	Rate constant for the degradation of Lin28	0.002
*V*_SLET7_	Maximum rate of synthesis of Let7 microRNA	3
*K*_ILET7_	Michaelis constant for the repression of Let7 synthesis by Lin28	0.1
*k*_1_	Bimolecular rate constant for binding of Let7 to mIL6	10
*k*_2_	Rate constant for dissociation of complex (mIL6Let7) between Let7 and mIL6	0.01
*k*_3_	Bimolecular rate constant for binding of Let7 to mRas	10
*k*_4_	Rate constant for dissociation of complex (mRasLet7) between Let7 and mRas	0.01
*k*_5_	Bimolecular rate constant for binding of miR21 to mPTEN	10
*k*_6_	Rate constant for dissociation of complex (miRmpten) between miR21 and mPTEN	0.01
*k*_DLET7_	Rate constant for degradation of Let7 microRNA	0.01
*V*_S1MIL6_	Independent rate of synthesis of IL6 mRNA, mIL6	0.1
*V*_S2MIL6_	Maximum rate of synthesis of IL6 mRNA depending on NFKB	0.01
*K*_A2NF_	Michaelis constant for the activation of mIL6 synthesis by NFKB	5
*k*_DMIL6_	Rate constant for the degradation of IL6 mRNA	0.01
*k*_DILLET_	Rate constant for the degradation of the complex between Let7 and mIL6	0.5
*k*_SIL6_	Rate constant for the synthesis of IL6 protein	1.2
*k*_DIL6_	Rate constant for the degradation of IL6 protein	0.1
*V*_SMRAS_	Rate of synthesis of Ras mRNA, mRas	0.005
*k*_DMRAS_	Rate constant for the degradation of mRas	0.01
*k*_DRASLET_	Rate constant for the degradation of the complex between Let7 and mRas	0.5
*k*_SRAS_	Rate constant for the synthesis of Ras protein	1
*k*_DRAS_	Rate constant for the degradation of Ras protein	0.1
*V*_SSTAT_	Maximum rate of synthesis of STAT3	0.5
*K*_AIL6_	Michaelis constant for the activation of STAT3 synthesis by IL6	40
*k*_DSTAT_	Rate constant for the degradation of STAT3	0.1
*V*_SMIR21_	Maximum rate of synthesis of miR21 microRNA	4
*K*_ASTAT_	Michaelis constant for the activation miR21 synthesis by STAT3	5
*k*_DMIR21_	Rate constant for the degradation of miR21	0.2
*V*_SMPTEN_	Rate of synthesis of PTEN mRNA, mPTEN	0
*k*_DMPTEN_	Rate constant for the degradation of mPTEN	0.01
*k*_DMIRMP_	Rate constant for the degradation of the complex between mPTEN and miR21	0.01
*k*_SPTEN_	Rate constant for the synthesis of PTEN protein	0.05
*k*_DPTEN_	Rate constant for the degradation of PTEN protein	0.1
**Addition of a competing endogenous RNA (ceRNA), which can bind to Let-7 microRNA**		
*V*_SCERNA_	Rate of synthesis of ceRNA	
*k*_7_	Bimolecular rate constant for binding of Let7 to ceRNA	10
*k*_8_	Rate constant for dissociation of complex (ceRNAlet7) between Let7 and ceRNA	0.01
*k*_DCERNA_	Rate constant for the degradation of ceRNA	0.01
*k*_DCELET7_	Rate constant for the degradation of the complex ceRNAlet7	0.01
**Addition of PTEN1 mRNA, which can bind to the microRNA miR-21**		
*V*_SMPTEN1_	Rate of synthesis of PTEN1 mRNA, mPTEN1	
*k*_9_	Bimolecular rate constant for binding of mPTEN1 to miR21	10
*k*_10_	Rate constant for dissociation of complex (miRmpten1) between mPTEN1 and miR21	0.01
*k*_DMPTEN1_	Rate constant for the degradation of mPTEN1	0.01
*k*_DMIRMP1_	Rate constant for the degradation of miRmpten1	0.01

Note: Because many of the parameters have not been determined experimentally, and since the study focuses on the dynamic implications of the regulatory structure of the epigenetic switch linking inflammation to cell transformation —i.e., its wiring diagram, or topology, which is crucial for the network behavior (see [65])— the numerical values have been selected so as to yield a non-transformed state without the inflammatory signal, Src; or a transformed state within 36 h–60 h depending on the duration of the transient inflammatory signal (see Fig. 2 as well as experimental observations in Ref. [7]). The time units for the parameters in the model are expressed in minutes, while the concentrations are expressed in µM.

The mechanisms of control of miRNA degradation are complex and not fully understood [66]. To keep the model simple, we hypothesize that each complex between a messenger RNA and its regulating miRNA is rapidly targeted for degradation.

Moreover, the rates of synthesis and degradation of messenger RNAs and proteins considered in the model are in the same order of magnitude as observed experimentally in a global quantification of mammalian gene expression control [67].

Finally, to compensate for our lack of knowledge about parameter values, we performed many bifurcation analyses, which bring to light the dynamical behaviors of the model as parameter values vary. In our experience, the dynamical properties of such models depend more on its network structure than on precise values of the parameters.

A description of the effect of a competing endogenous RNA, ceRNA, for Let-7 microRNA, as well as the effect of PTEN1 mRNA on the dynamics of the epigenetic switch can be found in subsection 2: “Effect of competing endogenous RNA, ceRNA, binding to Let-7 microRNA on the dynamics of the epigenetic switch leading to cell transformation”; and in subsection 3: “Role of PTEN1 mRNA in cell transformation”, respectively. The values for the initial conditions used in the simulations can be found in subsection 4: “Initial conditions”.

### 1. Kinetic equations of the model

In the model, we consider for simplicity a constant total concentration of NF-κB, *NFKB*_T_. Activation and inactivation reactions of NF-κB behave as Goldbeter-Koshland switches [64]. All other processes of the model rest on mass-action kinetics.
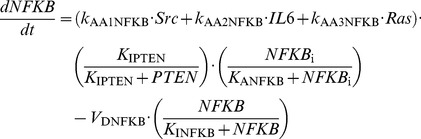
(1)
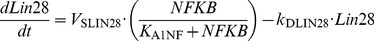
(2)
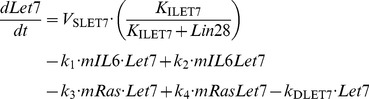
(3)
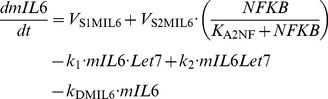
(4)
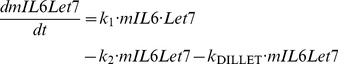
(5)
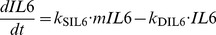
(6)
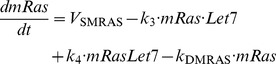
(7)
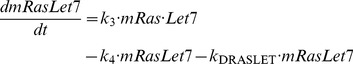
(8)
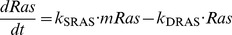
(9)
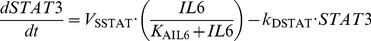
(10)
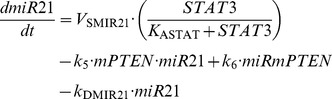
(11)
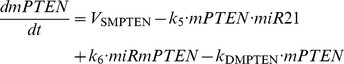
(12)
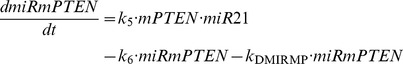
(13)
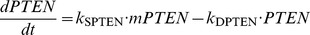
(14)With the following conservation equation:
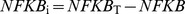
The ‘ode’ code of the deterministic model can be found in Supporting Information (Model S1). Such code can be run quite easily with the program XPP/XPPAUT (http://www.math.pitt.edu/~bard/xpp/xpp.html).

### 2. Effect of competing endogenous RNA, ceRNA, binding to Let-7 microRNA on the dynamics of the epigenetic switch leading to cell transformation

To account for the effect of a competing endogenous RNA, ceRNA, binding to Let-7 microRNA on the dynamics of the epigenetic switch, we add two kinetic equations describing the time evolution of ceRNA (see Eq. (15)) as well as the time evolution of the inhibitory complex between ceRNA and Let-7, ceRNAlet7 (see Eq. (16)). Moreover, we include the terms of association and dissociation of Let-7 to ceRNA in the kinetic equation describing the time evolution of Let-7 in Eq. (3) (see new Eq. (3′)).
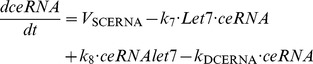
(15)
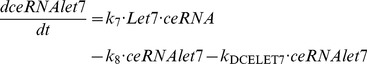
(16)
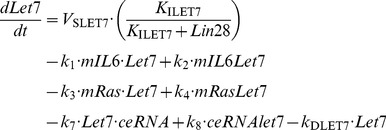
(3’)

### 3. Role of PTEN1 mRNA in cell transformation

In a similar manner, to account for the effect of PTEN1 mRNA, mPTEN1, on the dynamics of the epigenetic switch, we add two kinetic equations describing the time evolution of mPTEN1 (see Eq. (17)) as well as the time evolution of the inhibitory complex between mPTEN1 and the microRNA miR-21, miRmPTEN1 (see Eq. (18). Moreover, we include the terms of association and dissociation of miR-21 to mPTEN1 in the kinetic equation describing the time evolution of miR-21 in Eq. (11) (see new Eq. (11′)).
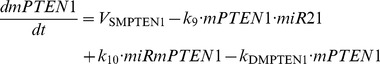
(17)
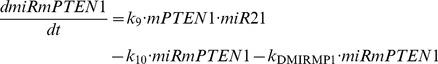
(18)
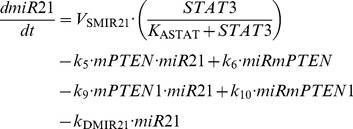
(11’)

### 4. Initial conditions

In the simulations of Figs. 2 and S1, with the parameter values of Table 2 and with Src = 0, the following initial conditions have been chosen so as to reach a non-transformed state of the cell characterized by high levels of Let7 and PTEN together with low levels of NF-κB, Lin28, IL6 and STAT3.

*NFKB* = 0.01; *Lin28* = 0.01; *Let7* = 1; *mIL6* = 0.01; *IL6* = 0.01; *mRas* = 0.01; *Ras* = 0.01; *STAT3* = 0.01; *mPTEN* = 0.01; *mRasLet7* = 0; *mIL6Let7* = 0; *miR21* = 0; *PTEN* = 0; *MiRmPTEN* = 0.

For the same conditions on the parameters, the following initial conditions have been chosen in all the other figures, giving rise to a switch at about t = 20 h.

*NFKB* = 0.5; *Lin28* = 0.5; *Let7* = 1; *mIL6* = 0.5; *IL6* = 0.5; *mRas* = 0.5; *Ras* = 0.5; *STAT3* = 0.5; *mPTEN* = 0.01; *mRasLet7* = 0; *mIL6Let7* = 0; *miR21* = 0; *PTEN* = 0; *MiRmPTEN* = 0; CeRNA = 0; *CeRNALet7* = 0; *mPTEN1* = 0; *MiRmPTEN1* = 0.

Because the dynamics of the model for the epigenetic switch linking inflammation to cell transformation rests on an irreversible bistable switch, such dynamics will be sensitive to the value of the initial conditions of the different variables defining the system. For the same parameter values, depending on the values of the initial conditions, the system may tend to a transformed or a non-transformed state of the cell (not shown).

## Supporting Information

Figure S1 Importance of the positive inflammatory feedback loop to maintain the transformed state of the cell. Time evolution of NF-κB, Lin28, Let-7, IL6 and STAT3 is shown in the presence of transient inhibition of NF-κB (A), Lin28 (B), or IL6 (C). In each case, from t = 0, 5 minutes of Src signaling is sufficient to trigger cell transformation at t = 60 h (same condition as in Fig. 2C). From 100 h<t<130 h, a transient inhibition of NF-κB (*k*_AA1NFKB_ = *k*_AA2NFKB_ = *k*_AA3NFKB_ = 0) in A, of Lin28 (*V*_SLIN28_ = 0) in B, or of IL6 (*V*_S1MIL6_ = *V*_S2MIL6_ = 0) in C is sufficient to abolish the transformed state and to recover a normal, non-transformed state, characterized by a high level of Let-7 together with low levels of Lin28, IL6, NF-κB and STAT3. Other parameter values are as in Fig. 2C.
(TIF)

Figure S2 Effect of the tumor suppressor PTEN on the dynamical behavior of the switch linking inflammation to cell transformation. Steady-state levels of Let-7 and IL6 are represented as a function of Src in panels A and B respectively, for different rates of transcription of PTEN, *V*_SMPTEN_. Solid curves: stable steady states, dashed curves: unstable states. A non-transformed state of the cell is associated with high levels of Let-7 together with low levels of IL6, while low levels of Let-7 together with high levels of IL6 characterize a transformed state. By increasing *V*_SMPTEN_ from 0.01 to 0.5, the threshold for cell transformation moves to higher values of Src, which might correspond to experimental observations showing the tumor-suppressive properties of PTEN (see Ref. [47]). Parameter values are as in Fig. 3 with *V*_SLET7_ = 6 and *V*_SMRAS_ = 0.03.
(TIF)

Figure S3 Effect of PTEN on the robustness of the epigenetic switch towards stochastic fluctuations. Deterministic steady state levels of IL6 *vs* Src are shown for a low, *V*_SMPTEN_ = 0.01 in A, and for a high rate of synthesis of PTEN mRNA, *V*_SMPTEN_ = 0.05 in C. Red dots correspond to the stable steady state reached with the initial conditions used. The corresponding stochastic simulations illustrate the proportion of transformed *vs* non-transformed cells as a function of Src in B and D. The proportion of cells is calculated with 50 stochastic cells for each condition (see also Figs. 6 and 7). An increase in the level of PTEN enhances the robustness of the switch towards stochastic fluctuations by decreasing the proportion of transformed cells at low values of Src (compare B and D). Parameter values are as in Table 2 with *V*_SLET7_ = 3.5.
(TIF)

Figure S4 Robustness of the epigenetic switch leading to cell transformation in a heterogeneous cell population. Expression levels of Let-7 and IL6 are represented in normal, non-transformed conditions (see conditions of Fig. 8A and panels A, D, G, J); in presence of high levels of Ras (see conditions of Fig. 8C and panels B, E, H, K); and in the presence of high levels of Ras together with high levels of PTEN (see conditions of Fig. 8F and panels C, F, I, L). For each case, random variation of 5% (A, B, C), 10% (D, E, F), 25% (G, H, I), and 50% (J, K, L) are applied on every parameters of the model. For each simulation, 100 cells are considered. Other parameter values are as in Fig. 8.
(TIF)

Figure S5 Digital, on-off response of NF-κB in a heterogeneous cell population. Expression levels of Let-7 and NF-κB are illustrated for increasing levels of the inflammatory signal, Src. Src is equal to 0.00001 in A, 0.0001 in B, and 0.001 in C. In each condition, 25% of random variation on every parameter value of the model is applied. For each simulation, 100 cells are considered. Other conditions are as in Fig. 8A.
(TIF)

Figure S6 Role of miR-21 for the dynamics of cell transformation in a heterogeneous cell population. miR-21 *vs* STAT3 levels as well as miR-21 *vs* PTEN levels are represented in the presence of a low, *V*_SMIR21_ = 4 in A, B, and a high rate of synthesis of miR-21, *V*_SMIR21_ = 4000 in C, D; as well as in the presence of a high rate of synthesis of miR-21, *V*_SMIR21_ = 4000, together with a low rate of synthesis of Lin28, *V*_SLIN28_ = 0.008 (E, F). As observed in the experiments (see Ref. [20]), the expression levels of miR-21 and STAT3 are positively correlated, while the expression levels of miR-21 and PTEN are negatively correlated. (A, B) A mixed population of non-transformed (low levels of miR-21 and STAT3 together with a high level of PTEN) and transformed cells (high levels of miR-21 and STAT3 together with a low level of PTEN) is present. (C and D) From the condition in A and B, an increase in *V*_SMIR21_ triggers the switch of nearly all cells in the population to a transformed state. (E and F) From condition in C and D, a reduction in the level of Lin28 destabilizes the positive inflammatory feedback loop, which brings back a large proportion of cells in the population to a non-transformed state (see also Ref. [20]). In each case, 100 cells are considered with 10% of random variation from the default value on all parameters. Other default parameter values are as in Fig. 9.
(TIF)

Figure S7 Effect of a ceRNA, which binds to Let-7 microRNA, on the dynamics of the switch leading to cell transformation. Steady-state levels of NF-κB, Lin28, Let-7, IL6, STAT3 and ceRNA as a function of the inflammatory signal, Src, are shown in panels A to F, respectively. For each case, different rates of synthesis of ceRNA, *V*_SCERNA_ are considered. Here again, an irreversible bistable behavior characterizes the epigenetic switch leading to cell transformation. Solid curves: stable steady states; dashed curves: unstable states. A progressive increase in *V*_SCERNA_ from 0 to 1 moves the threshold associated with cell transformation to smaller values of Src. When *V*_SCERNA_ is large (*V*_SCERNA_ = 2), only the transformed state of the cell persists regardless of the level of Src. Parameter values are as in Table 2 with *V*_SLET7_ = 6.
(TIF)

Figure S8 Effect of a ceRNA binding to Let-7 on the robustness of the epigenetic switch towards stochastic fluctuations. Deterministic steady state levels of IL6 *vs* Src are shown when *V*_SCERNA_ = 0, 0.1 and 0.15 in panels A, C and E, respectively. Red dots indicate the stable steady states reached with the initial conditions used. The corresponding stochastic simulations showing the proportion of non-transformed and transformed cells *vs* Src are illustrated in panels B, D and F. The proportion of cells is calculated with 50 stochastic cells for each condition. In B, the absence of ceRNA predicts a robust switch leading to cell transformation, where all cells are in a non-transformed state for a low level of Src. (D) With a low level of ceRNA, a small proportion of transformed cells is present even with a low level of Src; while with a higher level of ceRNA, the model predicts that a large proportion of cells is present in a transformed state regardless of the level of Src (F). Parameter values are as in Table 2 with *V*_SLET7_ = 6.
(TIF)

Figure S9 PTEN1 mRNA as a tumor suppressor (see Ref. [28]). Time evolution of NF-κB, IL6, miR-21 and PTEN are shown in panels A to D, respectively. In each case, different rates of transcription of PTEN1 mRNA, *V*_SMPTEN1_ are considered. With the conditions used, an inflammatory response leading to cell transformation occurs quickly when *V*_SMPTEN1_ = 0 (red curve in each case). Cell transformation is characterized by the rise in the levels of NF-κB, IL6 and miR-21, while the level of PTEN decreases. A small level of PTEN1 mRNA (blue curve with *V*_SMPTEN1_ = 0.15) delays the occurrence of cell transformation. In the latter case, the model clearly shows the biphasic regulation of IL6 expression, as observed in the experiments (see Ref. [7]). With larger levels of PTEN1 mRNA (green curve with *V*_SMPTEN1_ = 0.2 or orange curve with *V*_SMPTEN1_ = 0.5), the model exhibits only a transient inflammatory response (see transient peak of IL6), which does not lead to cell transformation. Parameter values are as in Table 2 with *V*_SLET7_ = 1.15, *V*_SMIR21_ = 10, *K*_IPTEN_ = 0.1, *V*_SMPTEN_ = 3, and *k*_SPTEN_ = 0.3.
(TIF)

Model S1 Code for the deterministic version of the model linking inflammation to cell transformation, which can be run with the program XPP/XPPAUT (http://www.math.pitt.edu/~bard/xpp/xpp.html).
(PDF)

Table S1 Stochastic version of the model linking inflammation to cell transformation.
(DOCX)
