# Supplementary figures and images for: A Model for the Epigenetic Switch Linking Inflammation to Cell Transformation: Deterministic and Stochastic Approaches
Source: PLoS Comput Biol. 2014 Jan 30;10(1):e1003455. doi: 10.1371/journal.pcbi.1003455 (PMC3907303; doi:10.1371/journal.pcbi.1003455)

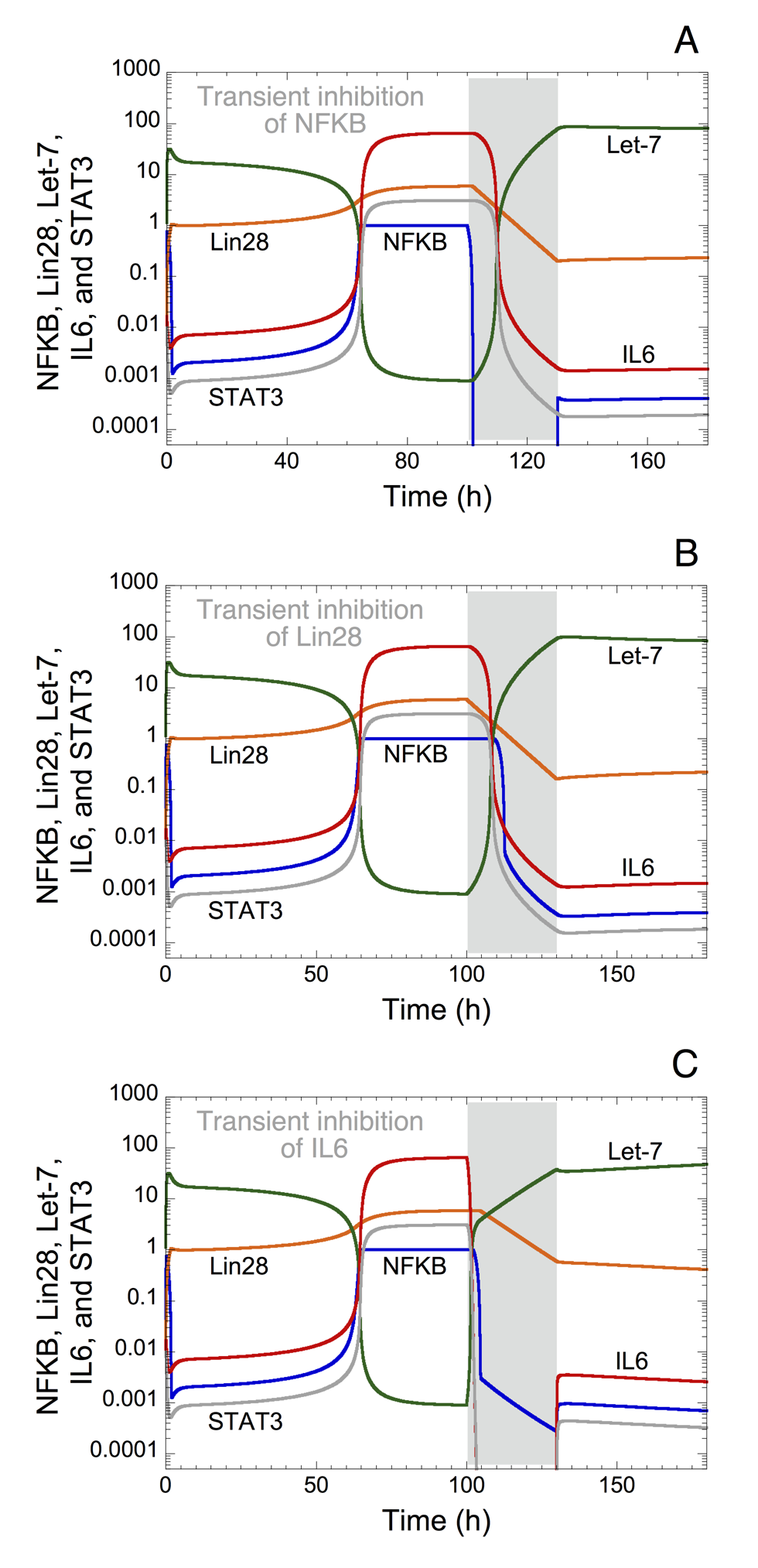

Supplement: Figure S1 — Importance of the positive inflammatory feedback loop to maintain the transformed state of the cell. Time evolution of NF-κB, Lin28, Let-7, IL6 and STAT3 is shown in the presence of transient inhibition of NF-κB (A), Lin28 (B), or IL6 (C). In each case, from t = 0, 5 minutes of Src signaling is sufficient to trigger cell transformation at t = 60 h (same condition as in Fig. 2C). From 100 h<t<130 h, a transient inhibition of NF-κB (kAA1NFKB = kAA2NFKB = kAA3NFKB = 0) in A, of Lin28 (VSLIN28 = 0) in B, or of IL6 (VS1MIL6 = VS2MIL6 = 0) in C is sufficient to abolish the transformed state and to recover a normal, non-transformed state, characterized by a high level of Let-7 together with low levels of Lin28, IL6, NF-κB and STAT3. Other parameter values are as in Fig. 2C. (TIF) [file pcbi.1003455.s001.tif]

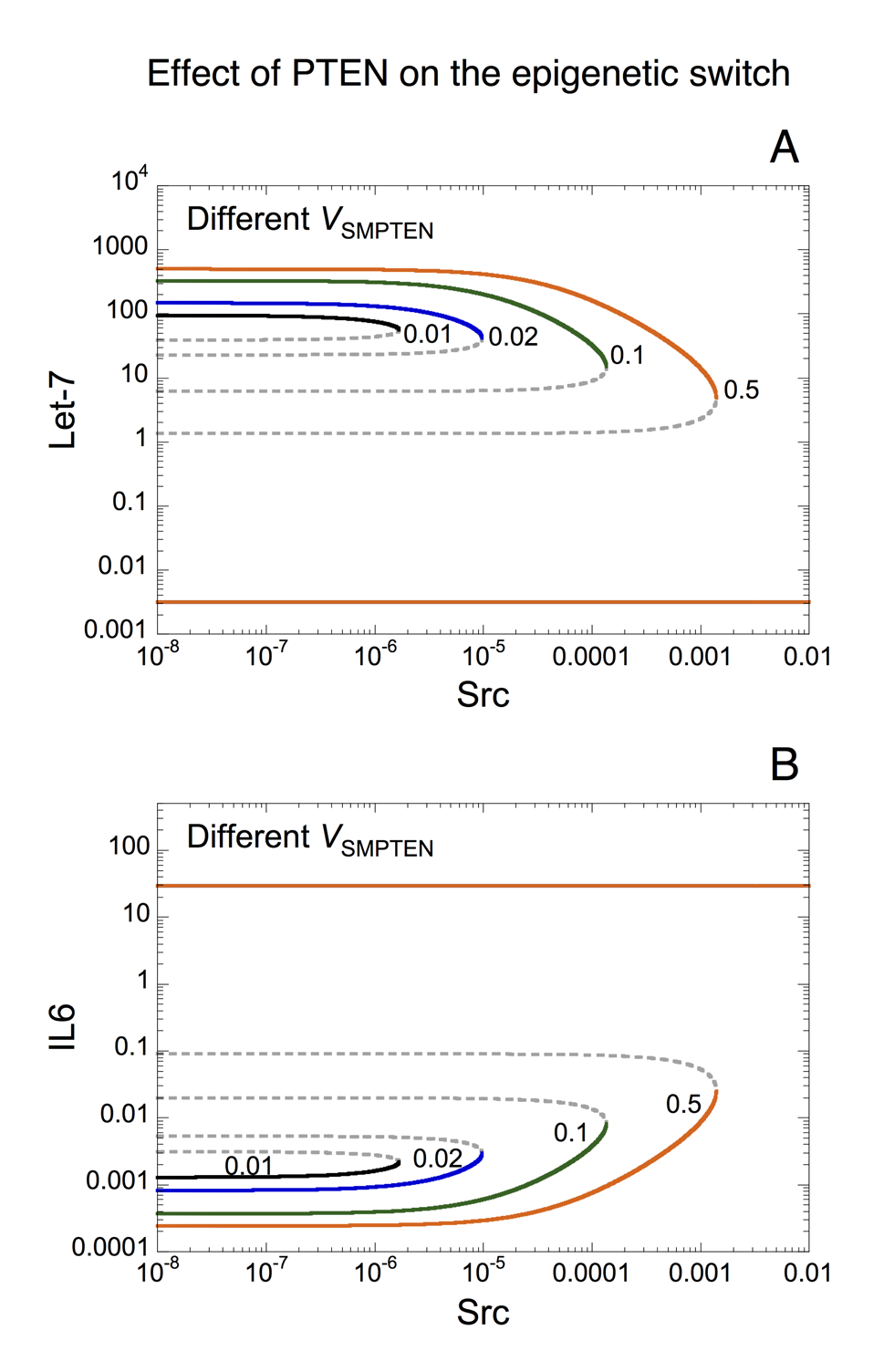

Supplement: Figure S2 — Effect of the tumor suppressor PTEN on the dynamical behavior of the switch linking inflammation to cell transformation. Steady-state levels of Let-7 and IL6 are represented as a function of Src in panels A and B respectively, for different rates of transcription of PTEN, VSMPTEN. Solid curves: stable steady states, dashed curves: unstable states. A non-transformed state of the cell is associated with high levels of Let-7 together with low levels of IL6, while low levels of Let-7 together with high levels of IL6 characterize a transformed state. By increasing VSMPTEN from 0.01 to 0.5, the threshold for cell transformation moves to higher values of Src, which might correspond to experimental observations showing the tumor-suppressive properties of PTEN (see Ref. [47]). Parameter values are as in Fig. 3 with VSLET7 = 6 and VSMRAS = 0.03. (TIF) [file pcbi.1003455.s002.tif]

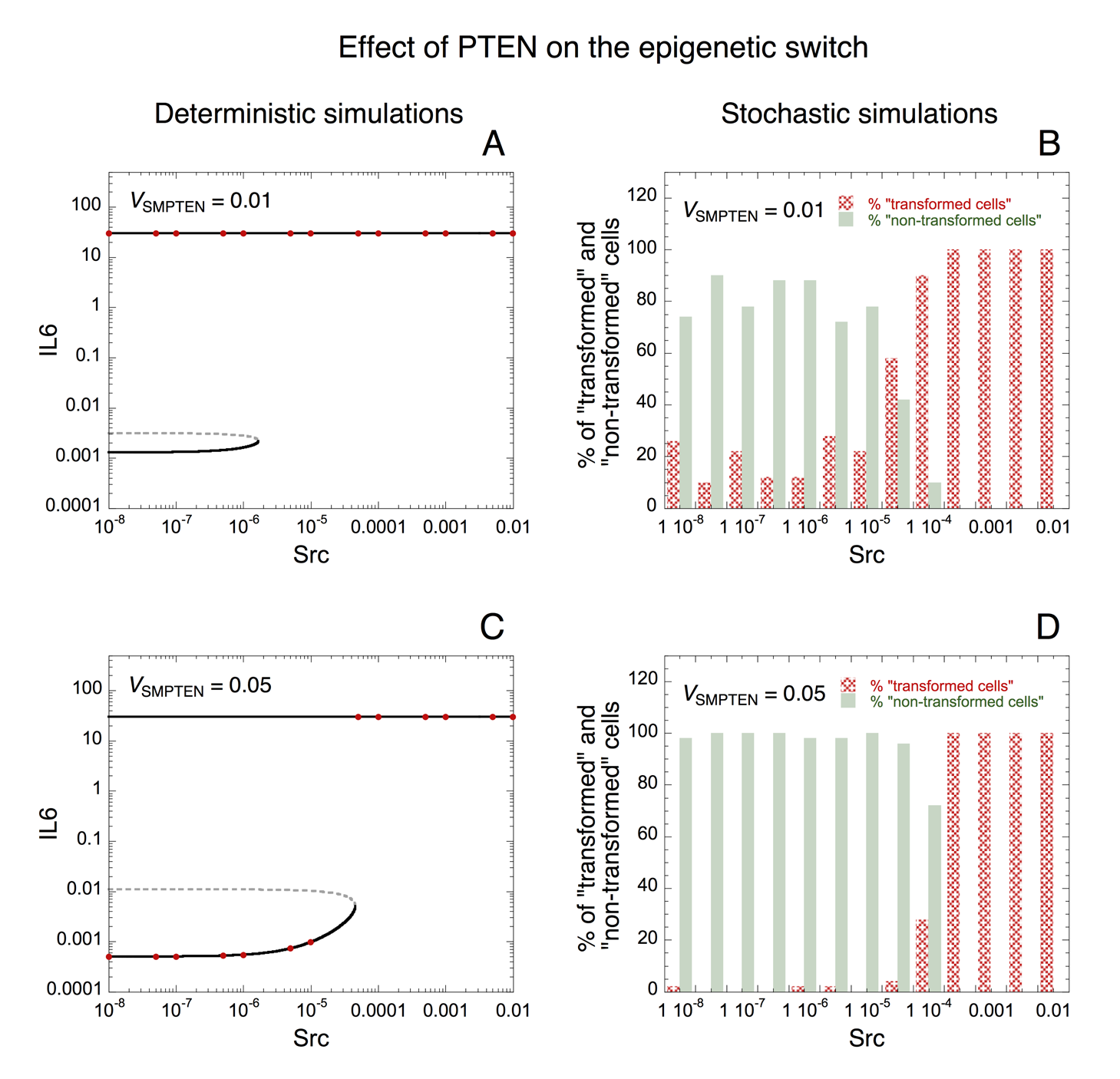

Supplement: Figure S3 — Effect of PTEN on the robustness of the epigenetic switch towards stochastic fluctuations. Deterministic steady state levels of IL6 vs Src are shown for a low, VSMPTEN = 0.01 in A, and for a high rate of synthesis of PTEN mRNA, VSMPTEN = 0.05 in C. Red dots correspond to the stable steady state reached with the initial conditions used. The corresponding stochastic simulations illustrate the proportion of transformed vs non-transformed cells as a function of Src in B and D. The proportion of cells is calculated with 50 stochastic cells for each condition (see also Figs. 6 and 7). An increase in the level of PTEN enhances the robustness of the switch towards stochastic fluctuations by decreasing the proportion of transformed cells at low values of Src (compare B and D). Parameter values are as in Table 2 with VSLET7 = 3.5. (TIF) [file pcbi.1003455.s003.tif]

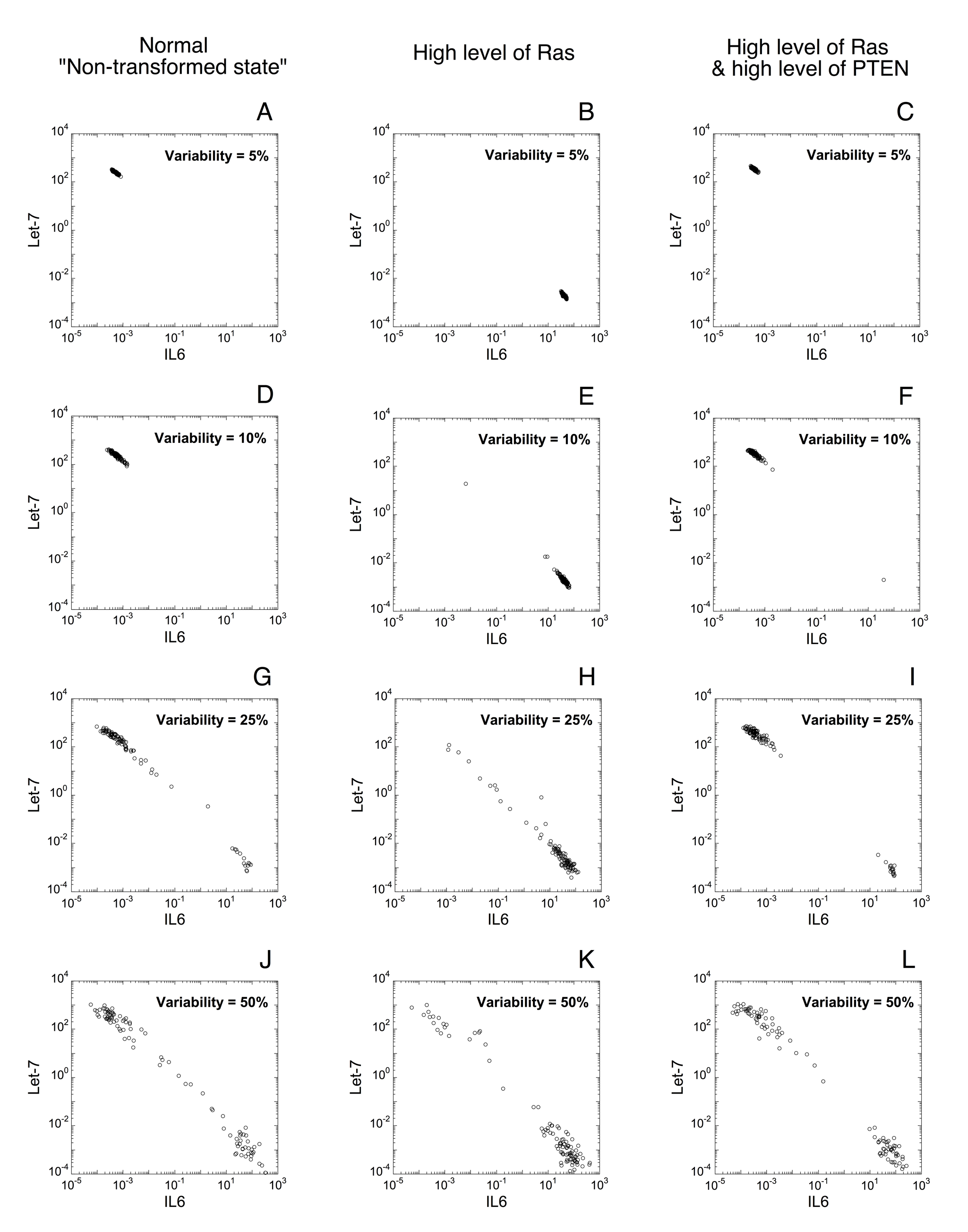

Supplement: Figure S4 — Robustness of the epigenetic switch leading to cell transformation in a heterogeneous cell population. Expression levels of Let-7 and IL6 are represented in normal, non-transformed conditions (see conditions of Fig. 8A and panels A, D, G, J); in presence of high levels of Ras (see conditions of Fig. 8C and panels B, E, H, K); and in the presence of high levels of Ras together with high levels of PTEN (see conditions of Fig. 8F and panels C, F, I, L). For each case, random variation of 5% (A, B, C), 10% (D, E, F), 25% (G, H, I), and 50% (J, K, L) are applied on every parameters of the model. For each simulation, 100 cells are considered. Other parameter values are as in Fig. 8. (TIF) [file pcbi.1003455.s004.tif]

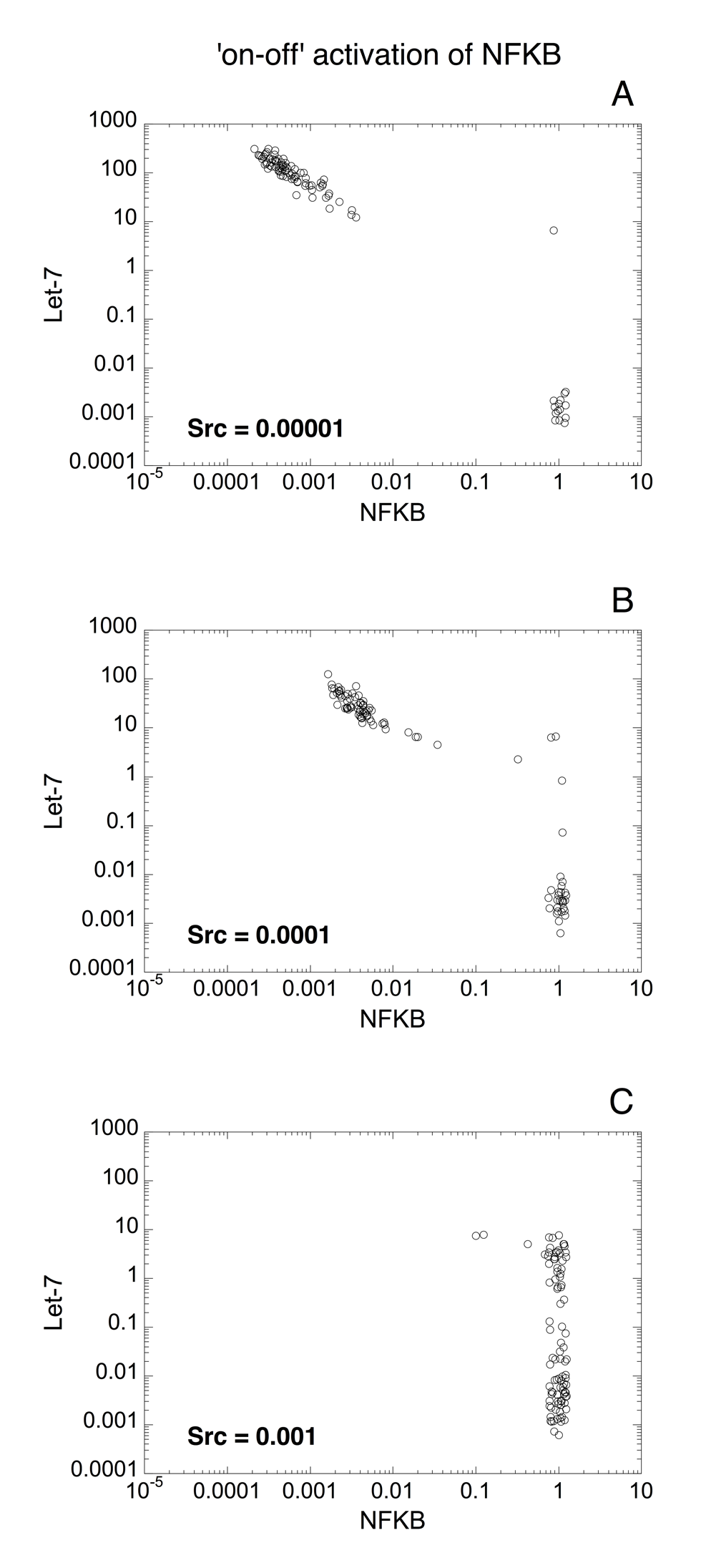

Supplement: Figure S5 — Digital, on-off response of NF-κB in a heterogeneous cell population. Expression levels of Let-7 and NF-κB are illustrated for increasing levels of the inflammatory signal, Src. Src is equal to 0.00001 in A, 0.0001 in B, and 0.001 in C. In each condition, 25% of random variation on every parameter value of the model is applied. For each simulation, 100 cells are considered. Other conditions are as in Fig. 8A. (TIF) [file pcbi.1003455.s005.tif]

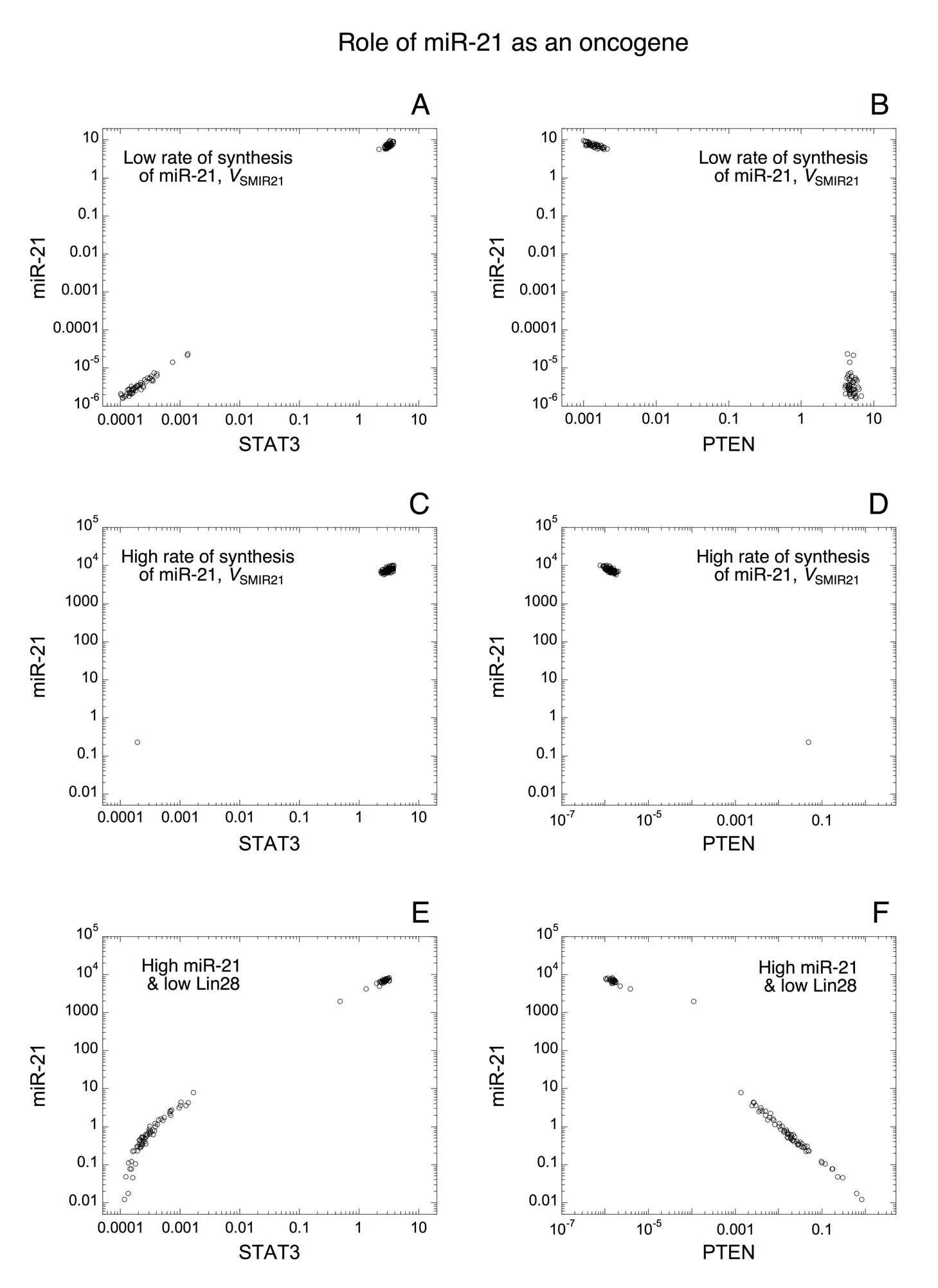

Supplement: Figure S6 — Role of miR-21 for the dynamics of cell transformation in a heterogeneous cell population. miR-21 vs STAT3 levels as well as miR-21 vs PTEN levels are represented in the presence of a low, VSMIR21 = 4 in A, B, and a high rate of synthesis of miR-21, VSMIR21 = 4000 in C, D; as well as in the presence of a high rate of synthesis of miR-21, VSMIR21 = 4000, together with a low rate of synthesis of Lin28, VSLIN28 = 0.008 (E, F). As observed in the experiments (see Ref. [20]), the expression levels of miR-21 and STAT3 are positively correlated, while the expression levels of miR-21 and PTEN are negatively correlated. (A, B) A mixed population of non-transformed (low levels of miR-21 and STAT3 together with a high level of PTEN) and transformed cells (high levels of miR-21 and STAT3 together with a low level of PTEN) is present. (C and D) From the condition in A and B, an increase in VSMIR21 triggers the switch of nearly all cells in the population to a transformed state. (E and F) From condition in C and D, a reduction in the level of Lin28 destabilizes the positive inflammatory feedback loop, which brings back a large proportion of cells in the population to a non-transformed state (see also Ref. [20]). In each case, 100 cells are considered with 10% of random variation from the default value on all parameters. Other default parameter values are as in Fig. 9. (TIF) [file pcbi.1003455.s006.tif]

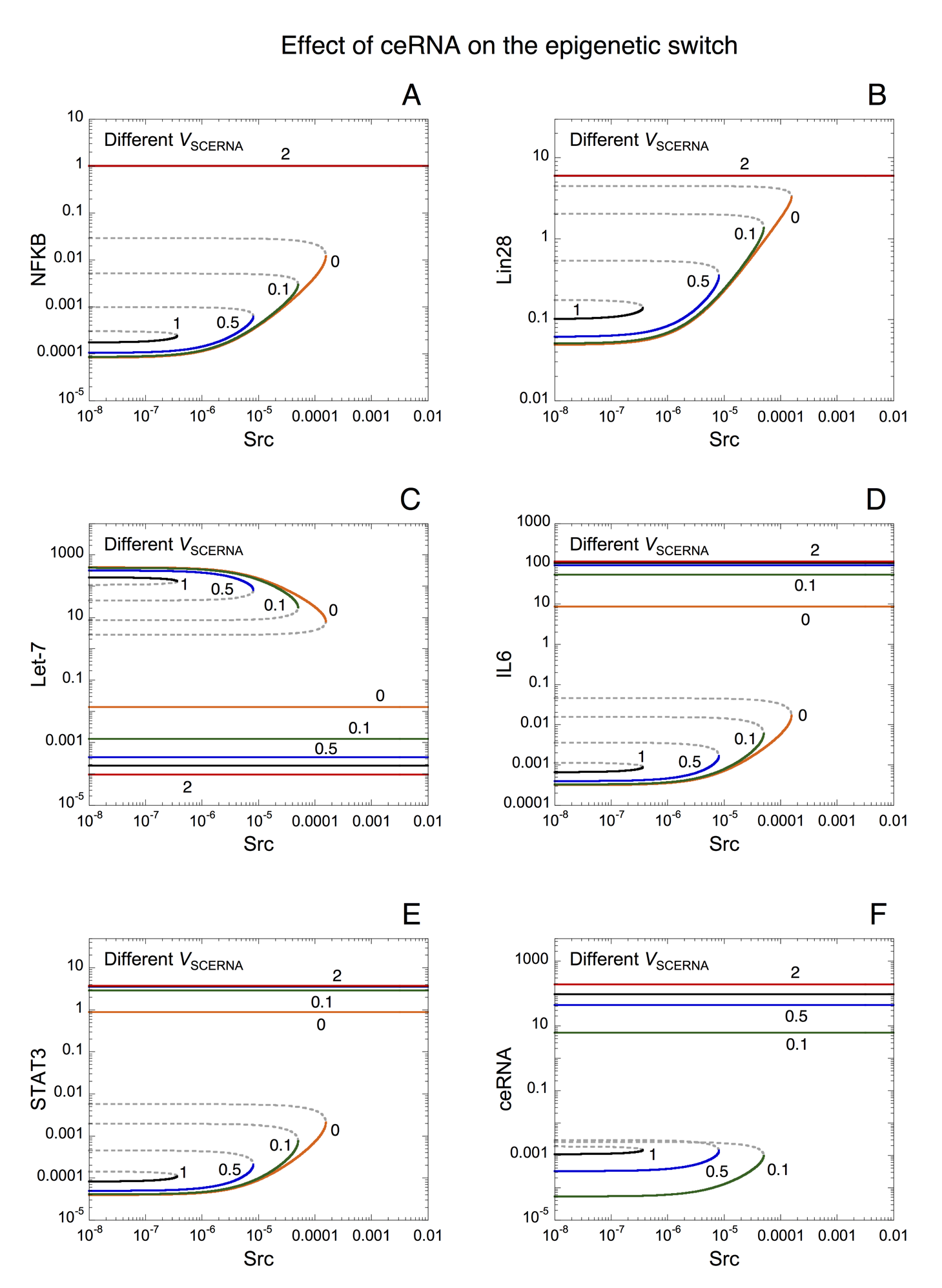

Supplement: Figure S7 — Effect of a ceRNA, which binds to Let-7 microRNA, on the dynamics of the switch leading to cell transformation. Steady-state levels of NF-κB, Lin28, Let-7, IL6, STAT3 and ceRNA as a function of the inflammatory signal, Src, are shown in panels A to F, respectively. For each case, different rates of synthesis of ceRNA, VSCERNA are considered. Here again, an irreversible bistable behavior characterizes the epigenetic switch leading to cell transformation. Solid curves: stable steady states; dashed curves: unstable states. A progressive increase in VSCERNA from 0 to 1 moves the threshold associated with cell transformation to smaller values of Src. When VSCERNA is large (VSCERNA = 2), only the transformed state of the cell persists regardless of the level of Src. Parameter values are as in Table 2 with VSLET7 = 6. (TIF) [file pcbi.1003455.s007.tif]

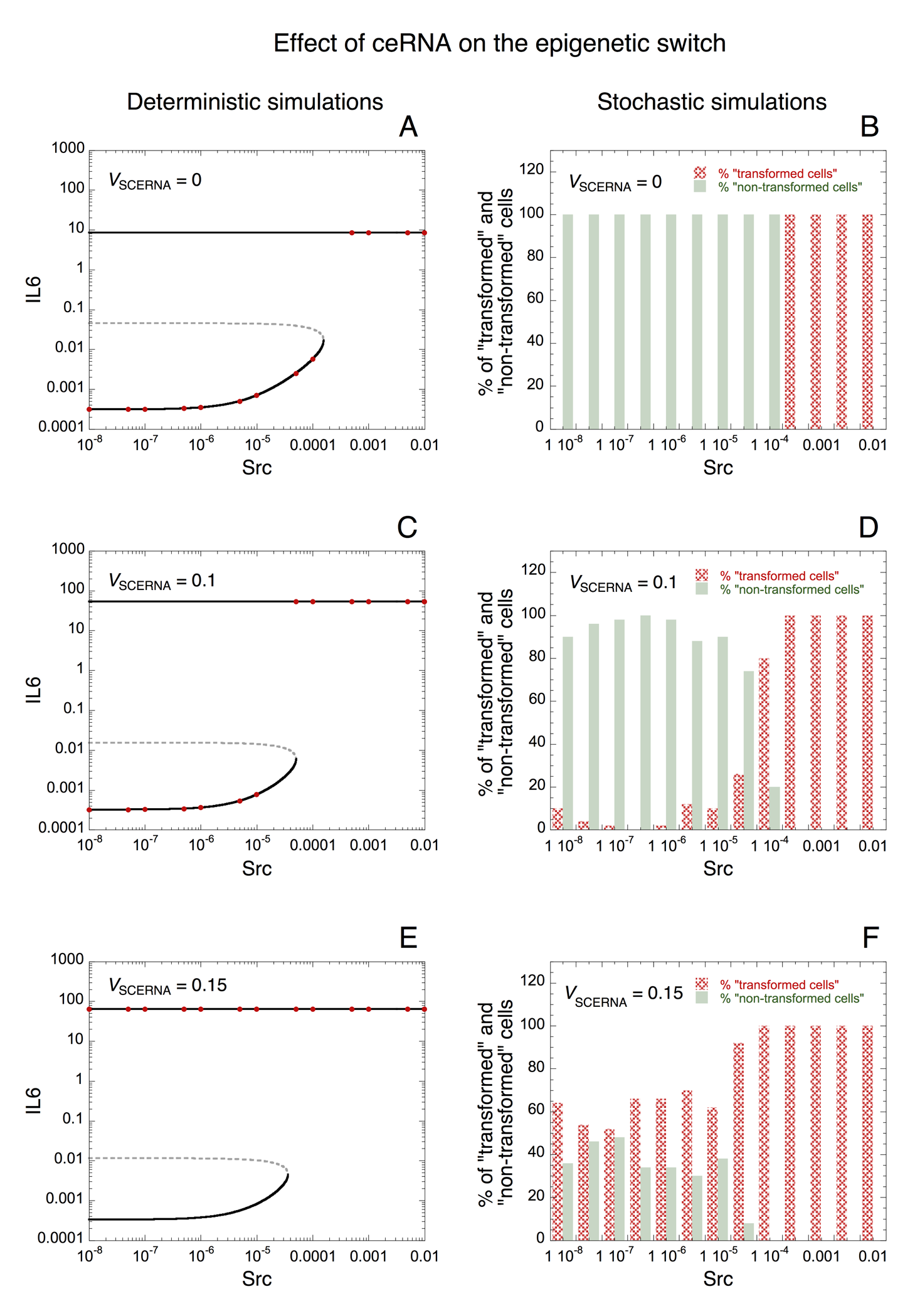

Supplement: Figure S8 — Effect of a ceRNA binding to Let-7 on the robustness of the epigenetic switch towards stochastic fluctuations. Deterministic steady state levels of IL6 vs Src are shown when VSCERNA = 0, 0.1 and 0.15 in panels A, C and E, respectively. Red dots indicate the stable steady states reached with the initial conditions used. The corresponding stochastic simulations showing the proportion of non-transformed and transformed cells vs Src are illustrated in panels B, D and F. The proportion of cells is calculated with 50 stochastic cells for each condition. In B, the absence of ceRNA predicts a robust switch leading to cell transformation, where all cells are in a non-transformed state for a low level of Src. (D) With a low level of ceRNA, a small proportion of transformed cells is present even with a low level of Src; while with a higher level of ceRNA, the model predicts that a large proportion of cells is present in a transformed state regardless of the level of Src (F). Parameter values are as in Table 2 with VSLET7 = 6. (TIF) [file pcbi.1003455.s008.tif]

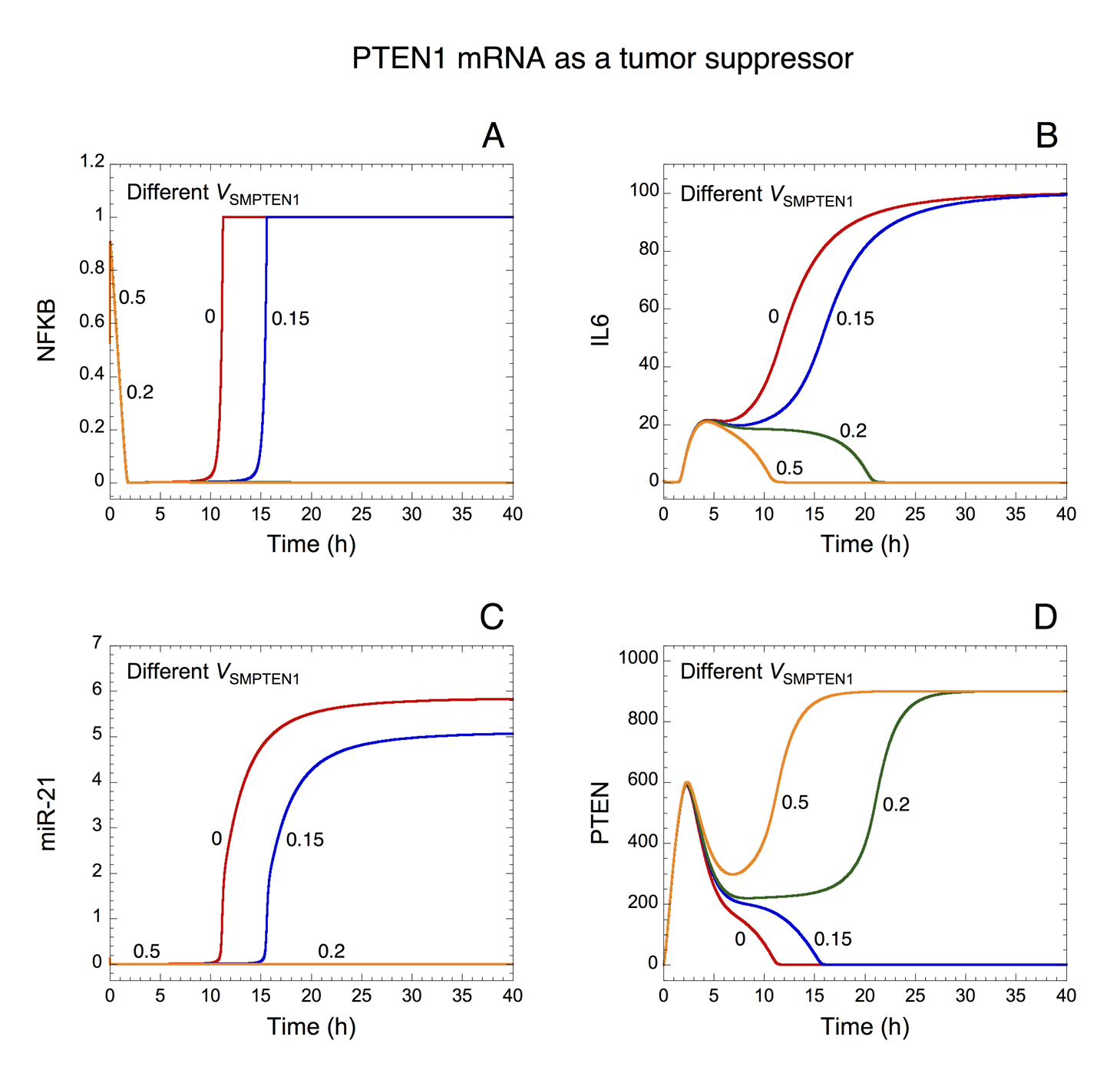

Supplement: Figure S9 — PTEN1 mRNA as a tumor suppressor (see Ref. [28]). Time evolution of NF-κB, IL6, miR-21 and PTEN are shown in panels A to D, respectively. In each case, different rates of transcription of PTEN1 mRNA, VSMPTEN1 are considered. With the conditions used, an inflammatory response leading to cell transformation occurs quickly when VSMPTEN1 = 0 (red curve in each case). Cell transformation is characterized by the rise in the levels of NF-κB, IL6 and miR-21, while the level of PTEN decreases. A small level of PTEN1 mRNA (blue curve with VSMPTEN1 = 0.15) delays the occurrence of cell transformation. In the latter case, the model clearly shows the biphasic regulation of IL6 expression, as observed in the experiments (see Ref. [7]). With larger levels of PTEN1 mRNA (green curve with VSMPTEN1 = 0.2 or orange curve with VSMPTEN1 = 0.5), the model exhibits only a transient inflammatory response (see transient peak of IL6), which does not lead to cell transformation. Parameter values are as in Table 2 with VSLET7 = 1.15, VSMIR21 = 10, KIPTEN = 0.1, VSMPTEN = 3, and kSPTEN = 0.3. (TIF) [file pcbi.1003455.s009.tif]
