# Supplementary material for: A Model for the Epigenetic Switch Linking Inflammation to Cell Transformation: Deterministic and Stochastic Approaches
Source: PLoS Comput Biol. 2014 Jan 30;10(1):e1003455. doi: 10.1371/journal.pcbi.1003455 (PMC3907303; doi:10.1371/journal.pcbi.1003455)
Supplement: Model S1 — Code for the deterministic version of the model linking inflammation to cell transformation, which can be run with the program XPP/XPPAUT (http://www.math.pitt.edu/~bard/xpp/xpp.html). (PDF) [file pcbi.1003455.s010.pdf]

```
### Model for the epigenetic switch linking inflammation to cell
transformation
### Gérard C., Gonze D., Lemaigre F., and Novak B.

### Model S1



# Initial conditions for Figures 2 and S1:
init NFKB=0.01, LIN28=0.01, let7=1, mIL6=0.01, IL6=0.01,
init mRas=0.01, Ras=0.01, STAT3=0.01, mPTEN=0.01,

# Initial conditions for other figures:
# init NFKB=0.5, LIN28=0.5, let7=1, mIL6=0.5, IL6=0.5, mRas=0.5, Ras=0.5,
STAT3=0.5, mPTEN=0,



# activation of Src
tm=mod(t,period)
tmtest=tm-period*X
forcing=1-heav(tmtest)
Src1=Srcmin+forcing*(Srcmax-Srcmin)
aux Src=Src1

# parameters
param period=10000, X=1, Srcmin=0, Srcmax=0,



# kinetic equations
dNFKB/dt=(kaa1nfkb*Src1 + kaa2nfkb*IL6 + kaa3nfkb*Ras)*(Kipten/(Kipten
+PTEN))*(NFKBi/(Kanfkb+NFKBi)) - Vdnfkb*(NFKB/(Kinfkb+NFKB))

dLIN28/dt=Vslin28*(NFKB/(Ka1nf+NFKB)) - kdlin28*LIN28

dlet7/dt=Vslet7*(Kilet7/(Kilet7+LIN28)) - k1*mIL6*let7 + k2*mIL6let7 -
k3*mRas*let7 + k4*mRaslet7 - kdlet7*let7

dmIL6/dt=Vs1mil6 + Vs2mil6*(NFKB/(Ka2nf+NFKB)) - k1*mIL6*let7 + k2*mIL6let7
- kdmil6*mIL6

dmIL6let7/dt=k1*mIL6*let7 - k2*mIL6let7 - kdillet*mIL6let7

dIL6/dt=ksil6*mIL6 - kdil6*IL6

dmRas/dt=Vsmras - k3*mRas*let7 + k4*mRaslet7 - kdmras*mRas

dmRaslet7/dt=k3*mRas*let7 - k4*mRaslet7 - kdraslet*mRaslet7
```

```
dRas/dt=ksras*mRas - kdras*Ras

dSTAT3/dt=ksstat*(IL6/(Kail6+IL6)) - kdstat*STAT3

dmiR21/dt=Vsmir21*(STAT3/(Kastat+STAT3)) - k5*mPTEN*miR21 + k6*miRmpten -
kdmir21*miR21

dmPTEN/dt=Vsmpten - k5*mPTEN*miR21 + k6*miRmpten - kdmpten*mPTEN

dmiRmpten/dt=k5*mPTEN*miR21 - k6*miRmpten - kdmiRmp*miRmpten

dPTEN/dt=kspten*mPTEN - kdpten*PTEN

NFKBi=NFKBT-NFKB



# parameters
param kaa1nfkb=10, kaa2nfkb=0.09, kaa3nfkb=1, Kanfkb=0.01, Kinfkb=0.02,
param Vdnfkb=0.01, NFKBT=1, Kasrc=2, Ka1il6=10, Kail6=40, Karas=10,
Kipten=5,
param Vslin28=0.012, kdlin28=0.002, Ka1nf=0.01,
param Vslet7=3, Kilet7=0.1, kdlet7=0.01,
param k1=10, k2=0.01, Vs1mil6=0.1, Vs2mil6=0.01, kdmil6=0.01, kdillet=0.5,
Ka2nf=5,
param ksil6=1.2, kdil6=0.1,
param Vsmras=0.005, k3=10, k4=0.01, kdmras=0.01,
param kdraslet=0.5, ksras=1, kdras=0.1,
param ksstat=0.5, kdstat=0.1,
param Vsmir21=4, Kastat=5, k5=10, k6=0.01, k7=10, k8=0.01, kdmir21=0.2,
param Vsmpten=0, kdmpten=0.01,
param kdmiRmp=0.01, kspten=0.05, kdpten=0.1,


@ Total=4500, meth=stiff, bound=10000, maxstor=1000000

done
```
