# Supplementary material for: A Model for the Epigenetic Switch Linking Inflammation to Cell Transformation: Deterministic and Stochastic Approaches
Source: PLoS Comput Biol. 2014 Jan 30;10(1):e1003455. doi: 10.1371/journal.pcbi.1003455 (PMC3907303; doi:10.1371/journal.pcbi.1003455)
Supplement: Table S1 — Stochastic version of the model linking inflammation to cell transformation. (DOCX) [file pcbi.1003455.s011.docx]

Table S1: Stochastic version of the model

| Reaction number | | Reaction | Propensity of reaction |
| --- | --- | --- | --- |
| 1 |  | |  |
| 2 |  | |  |
| 3 |  | |  |
| 4 |  | |  |
| 5 |  | |  |
| 6 |  | |  |
| 7 |  | |  |
| 8 |  | |  |
| 9 |  | |  |
| 10 |  | |  |
| 11 |  | |  |
| 12 |  | |  |
| 13 |  | |  |
| 14 |  | |  |
| 15 |  | |  |
| 16 |  | |  |
| 17 |  | |  |
| 18 |  | |  |
| 19 |  | |  |
| 20 |  | |  |
| 21 |  | |  |
| 22 |  | |  |
| 23 |  | |  |
| 24 |  | |  |
| 25 |  | |  |
| 26 |  | |  |
| 27 |  | |  |
| 28 |  | |  |
| 29 |  | |  |
| 30 |  | |  |
| 31 |  | |  |
| 32 |  | |  |
| 33 |  | |  |
| 34 |  | |  |
| **Addition of a competing endogenous RNA (ceRNA), which can bind to Let-7 microRNA** | | | |
| 35 |  | |  |
| 36 |  | |  |
| 37 |  | |  |
| 38 |  | |  |
| 39 |  | |  |
